# Synthetic sequence alignments as programmable probes of learned conformational landscapes in deep learning protein structure predictors

**DOI:** 10.1093/bioinformatics/btag605

**Published:** 2026-08-11

**Authors:** Jannik Adrian Gut, Noah Kleinschmidt, Thomas Lemmin

**Affiliations:** Institute of Biochemistry and Molecular Medicine, University of Bern, Bern 3012, Switzerland; Graduate School for Cellular and Biomedical Sciences (GCB), University of Bern, Bern 3012, Switzerland; Institute of Biochemistry and Molecular Medicine, University of Bern, Bern 3012, Switzerland; Graduate School for Cellular and Biomedical Sciences (GCB), University of Bern, Bern 3012, Switzerland; Institute of Biochemistry and Molecular Medicine, University of Bern, Bern 3012, Switzerland

## Abstract

**Motivation:**

Proteins rely on conformational flexibility for biological function, yet predicting alternative states remains a major challenge in structural biology. Although deep learning models like AlphaFold2, AlphaFold3, and RoseTTAFold2 excel at static structure prediction, what these networks actually learn about the underlying conformational landscapes remains largely opaque.

**Results:**

Here we introduce synthetic multiple sequence alignments (MSAs), designed by inverse folding to encode predefined structural constraints, as a programmable intervention for interrogating the internal logic of structure prediction systems. Synthetic MSAs systematically bias AlphaFold2, AlphaFold3, and RoseTTAFold2 toward distinct conformational states of fold-switching proteins, including alternative conformations inaccessible through natural sequence information alone. Adversarial experiments pairing query sequences with MSAs encoding competing folds reveal sequence-dependent responses, exposing how alignment-derived and sequence-derived signals are weighted within each system. Probing predictions initialized from molecular dynamics trajectories reveals a systematic bias toward compact, training-distribution-favored conformations. Hybrid alignments combining synthetic and natural MSA segments enable targeted steering toward specific conformational states. These results suggest synthetic MSAs as a generalizable framework for dissecting the conformational landscapes encoded by deep learning structure predictors, with direct implications for understanding model behavior and accessing biologically relevant hidden states.

**Availability and implementation:**

Newly generated data can be found at https://zenodo.org/records/20916910. The code underlying this article is available on GitHub at https://github.com/ibmm-unibe-ch/msa-tests.

## 1 Introduction

Proteins are dynamic molecular machines whose biological functions often rely on their ability to adopt multiple distinct conformations. This structural plasticity underpins essential processes, such as enzyme catalysis, molecular recognition, and allosteric regulation. Predicting this conformational diversity remains a major goal in structural biology, with implications for understanding disease mechanisms and advancing rational drug design.

In recent years, machine learning models, such as AlphaFold 2 (Jumper *et al.* 2021), AlphaFold 3 (Abramson *et al.* 2024), Boltz-2 (Passaro *et al.* 2025), Chai-1 team *et al.* or RoseTTAFold2 (Baek *et al.* 2023) have dramatically advanced the accuracy of protein structure prediction, establishing themselves as indispensable tools for elucidating protein function and guiding protein design. However, these models typically predict a single or small set of highly similar conformations for a given input sequence, and thus usually fail to capture the range of alternative states that many proteins exhibit *in vivo* (Wallner 2023, Masters *et al.* 2025). This limitation restricts their usage for studying mechanisms that depend on structural flexibility or fold switching.

State-of-the-art protein structure prediction pipelines generally consist of two key steps: (i) generating informative input representations of the input sequence and (ii) applying a deep learning model to predict atomic coordinates. Depending on the model, the input can range from a single protein sequence, as used in protein language model (pLM)-based approaches, such as OmegaFold (Wu *et al.* 2022) and ESMFold (Lin *et al.* 2023), to more complex multiple sequence alignments (MSAs) and structural templates, as required by AlphaFold (Jumper *et al.* 2021, Abramson *et al.* 2024) and RoseTTAFold2 (Baek *et al.* 2023). For MSA-based predictors, these alignments and templates are typically assembled by searching large sequence and structure databases (Berman *et al.* 2000, Suzek *et al.* 2015, Richardson *et al.* 2023, The UniProt Consortium 2025) with homology search tools, such as JackHMMER (Johnson *et al.* 2010), HHblits (Remmert *et al.* 2011), or MMseqs2 (Steinegger and Söding 2017).

Current strategies to increase conformational diversity focus either on modifying the machine learning model [e.g. through stochastic dropout or alternative network configurations (Wallner 2023)] or manipulating the MSA input. The latter approach, including subsampling (Del Alamo *et al.* 2022, Lee *et al.* 2025) or cluster-based selection (Wayment-Steele *et al.* 2024, Piomponi *et al.* 2025), has shown promise in producing multiple plausible states for some proteins. However, recent analyses indicate that MSA-based methods alone are insufficient to robustly capture fold switching or large-scale conformational changes (Chakravarty *et al.* 2023, 2024). Furthermore, the precise mechanism by which MSA-based methods steer the prediction and, critically, their inherent limitations, remain an active topic of research (Wayment-Steele *et al.* 2025).

In this study, we introduce synthetic MSAs, generated by inverse folding of target conformations, as a controlled experimental tool for probing information flow in deep learning structure predictors. We focus on AlphaFold2, AlphaFold3, and RoseTTAFold2 as representative MSA-based systems spanning current state-of-the-art performance. By encoding structural constraints directly into the input alignment, independently of natural homologs, we can systematically manipulate what these models receive and observe how they respond. We evaluate this approach on a benchmark set of fold-switching proteins (Porter and Looger 2018) previously used to expose limitations of AlphaFold (Chakravarty *et al.* 2024), and extend our analysis through adversarial sequence-MSA pairings and predictions initialized from long-timescale molecular dynamics trajectories. Together, these experiments reveal how alignment-derived and sequence-derived signals are weighted within each predictor, expose biases toward training-distribution-favored conformations, and demonstrate that synthetic MSAs can surface biologically relevant hidden states. Rather than proposing a new prediction pipeline, this work establishes MSA engineering as a principled approach for interrogating the conformational landscapes of state-of-the-art structure prediction systems.

## 2 Materials and methods

### 2.1 Inverse-folded multiple sequence alignments

Since protein structures deposited in the PDB (Berman *et al.* 2000) often have missing residues, do not start their residue count at 1 or have small changes from the deposited sequence to the structure, we first pre-process the structure by predicting the input sequence with ColabFold (Mirdita *et al.* 2022) and the database structure as a template and without MSA. It has been shown that AlphaFold 2 (Jumper *et al.* 2021) keeps the same structure of the template when there is no MSA information available (Gut and Lemmin 2025). Importantly, the scoring measurement is always compared to the database structure, not this pre-predicted structure.

The full pre-processed structure then gets inverse folded 128 times, the amount of sequences AlphaFold 2 handles without relying on bunching them into “extra_msa,” using ProteinMPNN (Dauparas *et al.* 2022) with the higher temperature of 1 instead of the default 0.1, to promote a higher amount of diversity in sequences, which should be more informative (Rao *et al.* 2021). The structure prediction is done with default parameters without templates or other biomolecules for the whole sequence as a monomer. On a smaller scale, such a pipeline was proposed previously (Wayment-Steele *et al.* 2025), before our project was disclosed.

The folded structure then gets cut using pdb-tools (Rodrigues *et al.* 2018) and compared to the database structure with OpenStructure (Biasini *et al.* 2013).

### 2.2 AlphaFold 2 cluster benchmark

Due to limited computational power, we constrained AlphaFold 2 Cluster (Wayment-Steele *et al.* 2024) to at most 50 clusters per input sequence by selecting the matching DBSCAN (Schubert *et al.* 2017) ϵ parameter while clustering instead of the default method that selects the most clusters possible.

### 2.3 Molecular dynamics simulations

All molecular dynamics simulations were performed in the NPT ensemble using OpenMM Eastman *et al.* (2024). Protein structures were first solvated in a cubic water box with 25 Å of padding in each direction and neutralized with KCl ions. The a99SB-disp force field (Robustelli *et al.* 2018) was used for proteins and the TIP4P-D 1.6 model for water (Piana *et al.* 2015, Sarthak *et al.* 2023). Temperature was controlled using a Langevin thermostat, and pressure was maintained with a Monte Carlo barostat. Hydrogen-bond constraints were applied to all bonds involving hydrogen atoms.

#### 2.3.1 Gaussian accelerated molecular dynamics (GaMD)

To sample the conformational space of fold-switching proteins, we extracted the fold switching fragment and employed Gaussian Accelerated Molecular Dynamics (GaMD) (Copeland *et al.* 2022). Triplicate simulations of $2.5\cdot 10^{8}$ steps with a 2 fs timestep were performed, corresponding to 500 ns per replicate, from which 25 000 frames were sampled. Simulations were conducted at 310 K using standard OpenMM settings for pressure coupling, hydrogen-bond constraints, and a Langevin integrator with GaMD-upper-dihedral boost. All parameters were set according to the default configuration from the OpenMM-GaMD implementation.

For one target (2NNT|A), the GaMD protocol failed to execute successfully. In this case, conventional molecular dynamics was run with OpenMM for up to 500 ns.

#### 2.3.2 High-temperature simulations

To study the folding behavior of fast-folding proteins, we performed high-temperature simulations of NTL9, Protein G, and Villin. The initial structure were taken from the fast-folding molecular dynamics dataset (Lindorff-Larsen *et al.* 2011). Three independent replicas were run for each protein at 427 K, each for up to 250 ns. Each system was energy minimized for 5000 steps, followed by pre-equilibration with restraints on backbone atoms for 750 ps, prior to production simulations. All simulations were run with the same timestep, thermostat, barostat, and hydrogen-bond constraints as the GaMD simulations, without applying the GaMD boost.

## 3 Results

**Figure 1:**
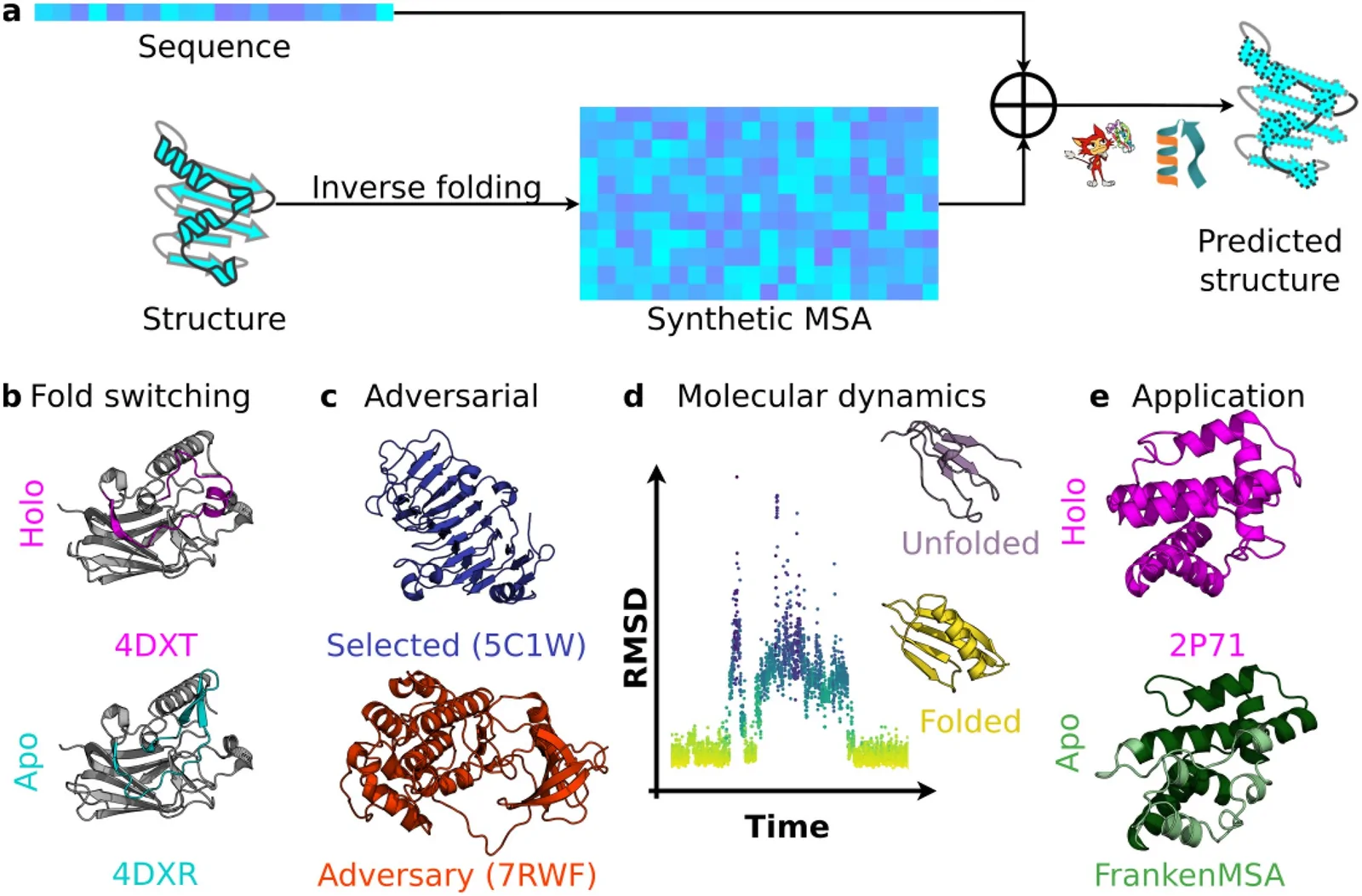
Overview of the proposed workflow and experimental use cases. (a) Schematic representation of the pipeline used throughout this study. Synthetic MSAs are generated from a protein structure, capturing its structural information, and are subsequently provided as input to a structure prediction model. Application of the pipeline to (b) a set of fold-switching proteins to evaluate reading out of alternative conformations, (c) assess sequence—MSA dependencies using adversarial MSAs, (d) examine the model’s learned structural distribution using molecular dynamics simulations and (e) construct of hybrid “*FrankenMSAs*” combining synthetic and natural MMseqs2 MSA segments to guide predictions toward specific structural states.

To probe how protein structure prediction models respond to controlled sequence alignment inputs, we developed a pipeline to generate synthetic MSAs by inverse folding from a given target structure (Fig. 1a, Section 4.1). The pipeline proceeds in two steps: (i) for each target structure, 128 sequences are generated using ProteinMPNN (Dauparas *et al.* 2022) conditioned on the input protein backbone coordinates; (ii) these sequences are compiled into a synthetic MSA, which is then provided as input to three structure prediction models: AlphaFold 2 (Jumper *et al.* 2021), AlphaFold 3 (Abramson *et al.* 2024), or RoseTTAFold2 (Baek *et al.* 2023).

### 3.1 Fold switching proteins

**Figure 2:**
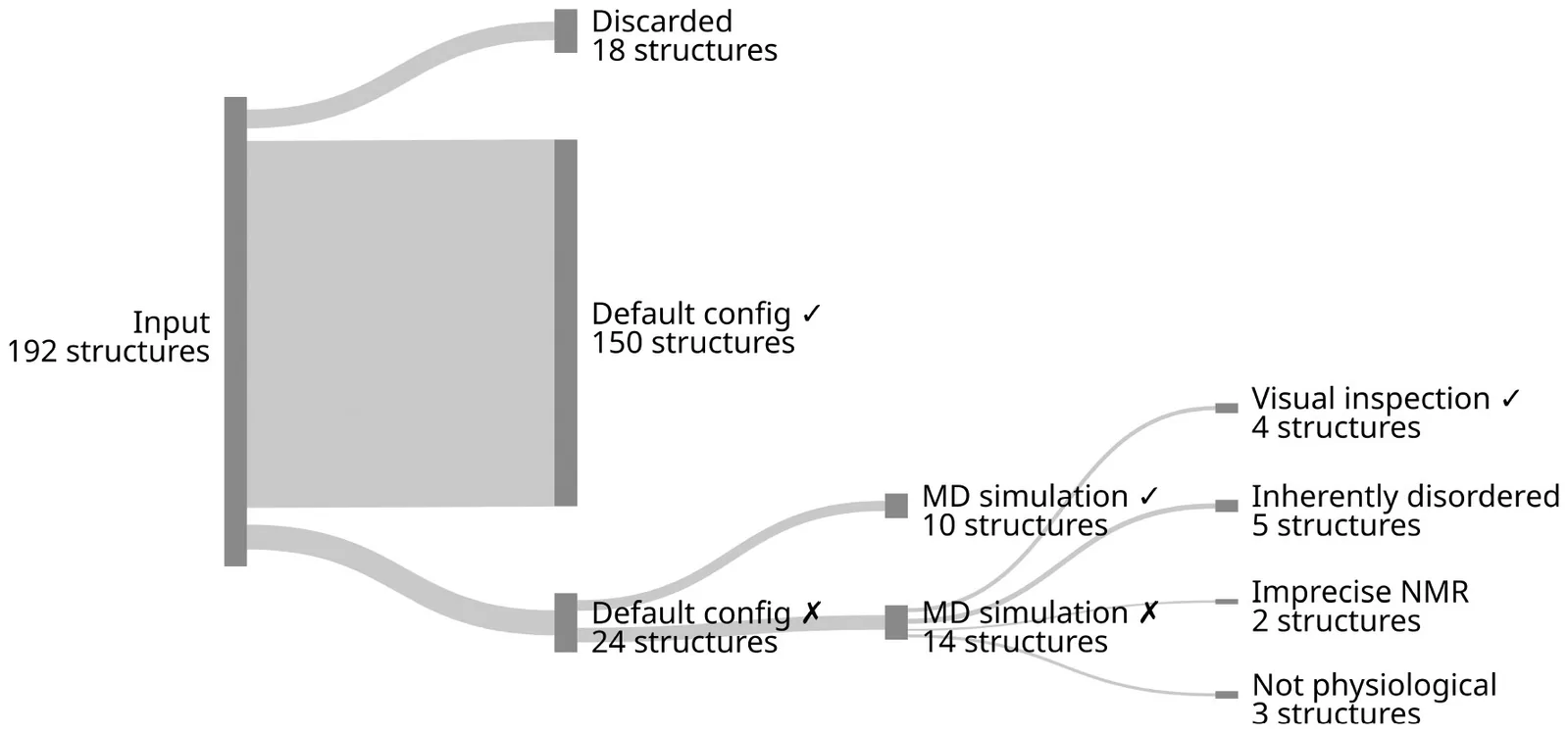
Sankey plot illustrating prediction outcomes for 192 starting structures in the fold-switching dataset. The leftmost column represents the starting structures, followed by the first phase showing prediction success using default configuration. The second phase depicts outcomes after MD simulations, and the rightmost column categorizes the structures that were not read out by the pipeline.

This pipeline was applied to a dataset of fold-switching proteins previously used to characterize gaps in AlphaFold 2’s ability to capture alternative conformations (Porter and Looger 2018, Chakravarty *et al.* 2023), providing a controlled setting in which to interrogate how synthetic MSAs modulate predictions across structurally distinct states. The original dataset comprises 96 pairs of distinct experimentally determined folds; after removing pairs with large differences in sequence length, obsolete experimental structures, or extensive unmodeled regions, 87 pairs remained for analysis. Sixteen of these were further truncated to remove residual unmodeled residues (details in Tables 1 and 2, available as supplementary data at *Bioinformatics* online). Prediction success was evaluated by comparing the predicted structures to the experimental folds using the local Distance Difference Test (lDDT) score (Mariani *et al.* 2013), with an average lDDT ≥ 0.7 considered a successful readout, consistent with established practice in protein structure assessment (Kovtun *et al.* 2024, Mulvaney *et al.* 2023, 2026). Using this criterion, the pipeline successfully read out 150 of 174 folds (Fig. 2).

Among the folds that could not be read out, we hypothesized that some experimental structures may represent metastable or non-native states due to crystal packing effects or conformational bias introduced during structure determination. To test this, we performed Gaussian accelerated Molecular Dynamics (GaMD) simulations on all unsuccessful targets to determine whether the experimental fold remains stable in solution or relaxes toward alternative conformations.

**Figure 3:**
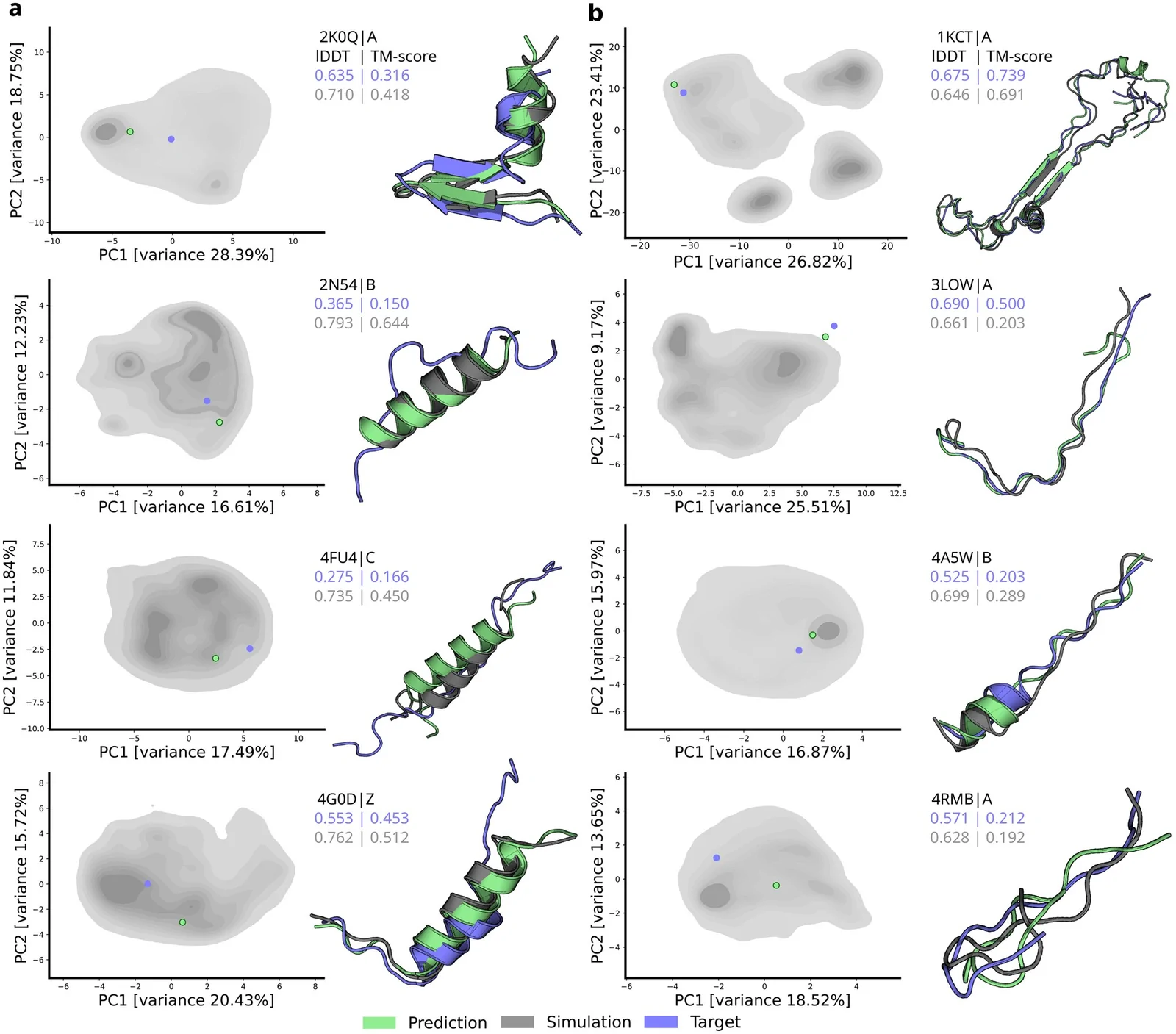
Representative structures obtained from molecular dynamics simulations with default parameters (a) and after manual inspection (b). PCA plots (left) depict the conformational space sampled during the GaMD simulations, projected onto the first two eigenvectors. lDDT and TM-scores comparing the prediction from the synthetic MSA to the initial target (blue) and to the closest simulation frame (gray) are reported. Cartoon representations show the closest simulation frame (gray), the target structure (blue), and the prediction (green) as indicated in the bottom legend.

For each simulation, we selected the five frames with the lowest backbone RMSD to the predicted structure after optimal superposition. These frames were treated as MD-sampled candidate conformations for the corresponding predicted fold. We then compared the synthetic-MSA predictions to these MD-refined frames to evaluate whether the predictions captured conformations accessible within the MD-sampled ensemble. This analysis revealed that, in ten cases, the predicted structures from the synthetic-MSA pipeline corresponded to conformations sampled during MD, indicating that these predictions capture states accessible when the experimental fold is relaxed through simulation (Fig. 3a and Fig. 2, available as supplementary data at *Bioinformatics* online).

We then examined the remaining 14 unsolved conformations. Four of these achieved TM-scores (Zhang and Skolnick 2005) greater than 0.5, indicating that key structural features were preserved despite not meeting the primary lDDT threshold. After visual inspection, we considered these four cases as partial successes and include them in the extended analysis of MD successes (Fig. 3b).

Among the 14 MD-solved structures (the ten previously solved plus the four partial successes), six proteins produced MD ensembles that overlapped with both the experimental structure and the synthetic-MSA prediction, placing the two structures within the same local region of conformational space (PDB: 1QLN|A, 2K0Q|A, 3O44|A, 4A5W|B, 4G0D|Z, 4RMB|A). One trajectory (1MBY|A) sampled a largely unstructured state. For the remaining seven structures, specific experimental or structural contexts likely contributed to deviations from the predicted fold: 4OW6|B represents the acid-induced conformational state of the diphtheria toxin, captured under low-pH conditions, and 4FU4|C is a peptide bound in the collagenase 3 catalytic pocket, which induces unfolding for enzymatic processing. Three additional structures required a binding partner (3J97|M, 3LOW|A) or involved an engineered binder (5K5G|A), reflecting inherent limitations of prediction models trained on isolated protein chains.

Of the remaining ten unsolved conformations, several present features that complicate their use as benchmarks for evaluating structure prediction models. In five cases (2AXZ|A, 2N0A|D, 2NAO|F, 5C1V|B, 5KEQ|F), the fold-switching fragments appear unfolded in the experimental data and are also predicted as unfolded (Fig. 1, available as supplementary data at *Bioinformatics* online). Although these regions have experimentally determined coordinates, they do not adopt a single, well-defined tertiary structure. Consequently, the reference conformation represents just one of many possible states, and discrepancies between predicted and reference structures in these regions should not be interpreted as model failures.

Two proteins (2NNT|A and 4HDD|A) required sequence modifications to stabilize the monomer during structure determination, suggesting that the wild-type forms may be intrinsically unstable. Another two targets (2JMR|A and 2LEL|A) were solved using NMR and show substantial variability across deposited models. To illustrate this, only 0/19 and 11/19 of the alternative NMR models for 2JMR|A and 2LEL|A, respectively, would meet our success threshold based on lDDT. In the case of 2JMR|A, the reference model failed, but another deposited model was successfully matched by our prediction. The final structure (2NAM|A) was determined in a micelle environment, which can promote non-physiological conformations and may not accurately reflect the protein’s native state.

These findings suggest that several of the remaining targets may involve conformations that are not well-defined, stable, or representative of native-like states. Such characteristics complicate their interpretation in the context of benchmarking the conformational sampling limitations of structure prediction models.

### 3.2 Performance breakdown and model comparisons

**Figure 4:**
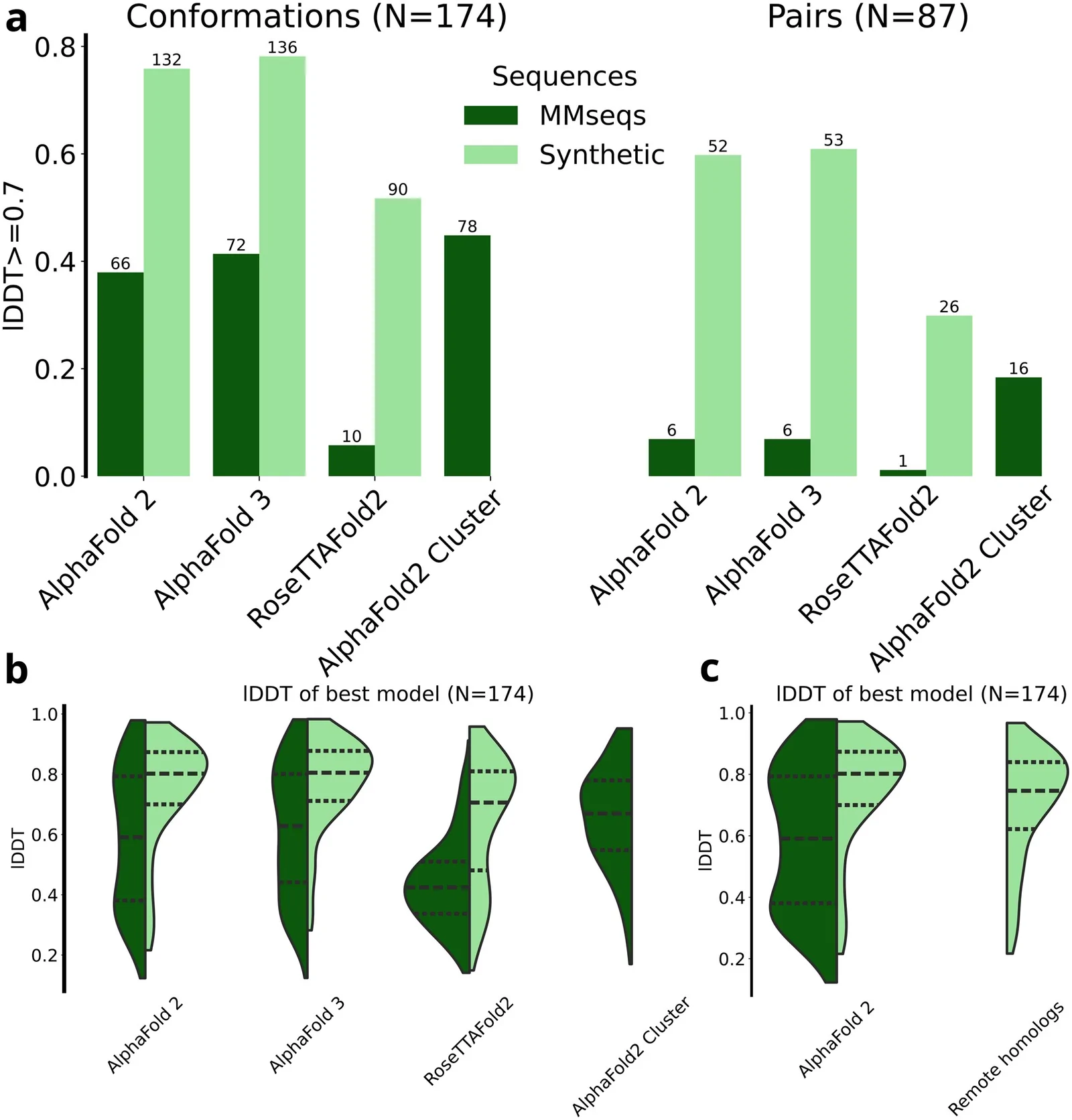
Performance of synthetic MSAs. (a) Ablation of different protein structure prediction models (AlphaFold 2, AlphaFold 3, RoseTTAFold2, AlphaFold 2 Cluster) as well as MMseqs2 and synthetic MSAs showing the amount of accepted conformations and conformation pairs, where both conformations were accepted. (b) Violin plot of lDDT scores of MMseqs2 MSAs, synthetic MSAs to compare different protein structure prediction models. (c) lDDT scores of MMseqs2 MSAs, synthetic MSAs and synthetic MSAs with close homologs removed.

We evaluated the performance of AlphaFold 2, AlphaFold 3, and RoseTTAFold2 using either our synthetic, inverse-folded MSAs or natural MSAs generated by the MMseqs2 web server (Mirdita *et al.* 2022). As expected, the synthetic MSAs significantly improve prediction accuracy across all models tested with *t*-test (Student 1908) *p*s lower than $10^{-5}$ (Table 3, available as supplementary data at *Bioinformatics* online). This improvement is consistent and structure prediction model-independent, indicating that our synthetic alignment strategy enhances the signal relevant to the target conformation while removing conflicting evolutionary noise (Fig. 4a and Fig. 3a and b, available as supplementary data at *Bioinformatics* online). In addition, the pLDDT scores remained consistently high across all models, with minimal variance across the ten models of AlphaFold 2 and AlphaFold 3 (Fig. 3c and d, available as supplementary data at *Bioinformatics* online), indicating that our pipeline produces stable and reliable structures without requiring extensive sampling or ensembling.

AlphaFold 3 slightly outperforms AlphaFold 2, in accordance with the reported marginal improvements in monomer structure accuracy (Abramson *et al.* 2024). RoseTTAFold2 shows the largest relative improvement from synthetic MSAs but does not reach the absolute performance levels of the AlphaFold-based models. This observation is consistent with the interpretation of protein structure prediction as an MSA decoding problem; weaker models benefit more from carefully curated MSAs that reduce ambiguity arising from conformational heterogeneity of aligned sequences.

We also evaluated AlphaFold 2 Cluster (Wayment-Steele *et al.* 2024), which we configured to run at most 50 parallel predictions per target to improve sampling. While it performs better than AlphaFold 2 and AlphaFold 3 with standard MMseqs2 MSAs, its accuracy still falls short of predictions generated with synthetic MSAs. In many cases, not all 50 prediction runs are utilized due to shallow MMseqs2 MSAs, limiting the effectiveness of extensive sampling.

We identified several conformations that were read out by only a single model: one by AlphaFold 2, seven by AlphaFold 3, and two by AlphaFold 2 Cluster, while none were uniquely read out by RoseTTAFold2. Unique recoveries are listed in Table 4, available as supplementary data at *Bioinformatics* online. Additionally, four conformations were only correctly predicted when using MSAs generated via the MMseqs2 web server. Closer examination reveals that three of these proteins (1NQD|A, 2LV1|A, 4YDQ|B) involve unfolded regions or predictions close to the lDDT threshold, where small stochastic differences, such as variations in random seed, likely pushed the models just above the success threshold. The fourth protein (2KKW|A) corresponds to alpha-synuclein in an SLAS-micelle environment, which may induce non-native conformations. We note that these borderline cases highlight the inherent variability of unfolded regions and the difficulty in assigning binary success labels to them, rather than indicating ineffectiveness of synthetic MSAs.

The notably lower number of successful sequence pair predictions, compared to the larger number of recovered individual conformations, can be attributed to the way the dataset was constructed. Specifically, the dataset was curated to identify similar sequences adopting distinct conformations. In many cases, one conformation is relatively easy to predict, while the alternative fold is more challenging. The presence of two such subpopulations is reflected in the bimodal distribution of lDDT scores when using MMseqs2-derived MSAs (Fig. 4b), whereas predictions using synthetic MSAs exhibit a more unimodal, shifted distribution, indicating improved consistency across both folds.

To assess the reliance of our approach on closely related sequences, we removed homologous sequences from the synthetic MSAs sharing over 30% sequence identity. This filtering led to a modest drop in performance, but still surpassed MSAs derived from MMseqs2 (Fig. 4c). These findings suggest that AlphaFold 2 does not depend solely on high-identity sequences or simple frequency statistics. Instead, it likely exploits more nuanced residue relationships, akin to substitution matrices like BLOSUM (Henikoff and Henikoff 1992) or more complex patterns embedded within the MSA and the input sequence.

In a more stringent ablation, we excluded all sequences from the synthetic MSA that could be matched to UniProt Consortium (2024) using MMseqs2’s *easy-search* (Steinegger and Söding 2017 ; The UniProt Consortium 2025). Of the 22 272 sequences generated across the dataset, only 38 had detectable matches at 60% sequence identity and 640 (3%) sequences had matches at 50% sequence identity, indicating that our synthetic MSAs are almost entirely composed of novel, non-natural sequences. Despite this, structure prediction performance remained robust, demonstrating that current models are not strictly dependent on the presence of real evolutionary sequences. This further supports the notion that synthetic MSAs can serve as a viable and generalizable input for guiding structure prediction, independent of sequence provenance.

To assess whether synthetic MSAs share statistical properties with MMseqs2-derived MSAs, we compared per-position entropy, gap fraction, and pairwise BLOSUM scores (Table 5, available as supplementary data at *Bioinformatics* online). Synthetic MSAs show higher per-position entropy (2.00 vs 1.48), no gaps, and lower pairwise BLOSUM scores (−0.50 vs −0.11), reflecting the absence of evolutionary pressure. Despite these differences, per-position entropy profiles correlate weakly but significantly between natural and synthetic MSAs (mean Spearman r=0.30), consistent with structurally constrained positions allowing less sequence variability in both cases. Finally, we evaluated four proteins with structures deposited after the AF2 training cutoff (Del Alamo *et al.* 2022, Chakravarty *et al.* 2024) and observed consistent results (Tables 6–10, available as supplementary data at *Bioinformatics* online), suggesting that performance is not solely driven by training set memorization, although memorization via homologous sequences cannot be fully ruled out.

### 3.3 Sequence-dependent response to adversarial MSAs

The strong performance of our pipeline raises a critical concern: can AlphaFold 2 be manipulated to predict arbitrary structures, provided the input MSA is engineered to support a given fold? If so, this would suggest an overreliance on MSA-derived signals, potentially undermining the model’s ability to generalize beyond tailored inputs. To explore this concern, we designed an adversarial test involving two datasets of structurally dissimilar protein pairs. Each dataset consists of protein pairs, one *selected* protein and one *adversarial* protein that share the same sequence length but otherwise differ substantially in both sequence identity and secondary structure. Additionally, all structures are fully solved, contain all residues, and have no engineered point mutations for structure determination to ensure comparability.

The first dataset includes 12 small, well-characterized *fast-folding* proteins (Lindorff-Larsen *et al.* 2011), which are often used in folding kinetics studies due to their simplicity and speed of folding. The second dataset consists of 30 structurally *diverse* proteins: they are longer, topologically varied, and selected to represent a broader range of fold space (Table 11, available as supplementary data at *Bioinformatics* online).

Since these experiments involve comparing protein structures with entirely different sequences and atomic compositions, lDDT is not an appropriate metric. Instead, we use the TM-score (Zhang and Skolnick 2005), which measures structural similarity based on backbone alignment and is sequence-independent. A TM-score above 0.5 is generally considered indicative of meaningful structural similarity.

**Figure 5:**
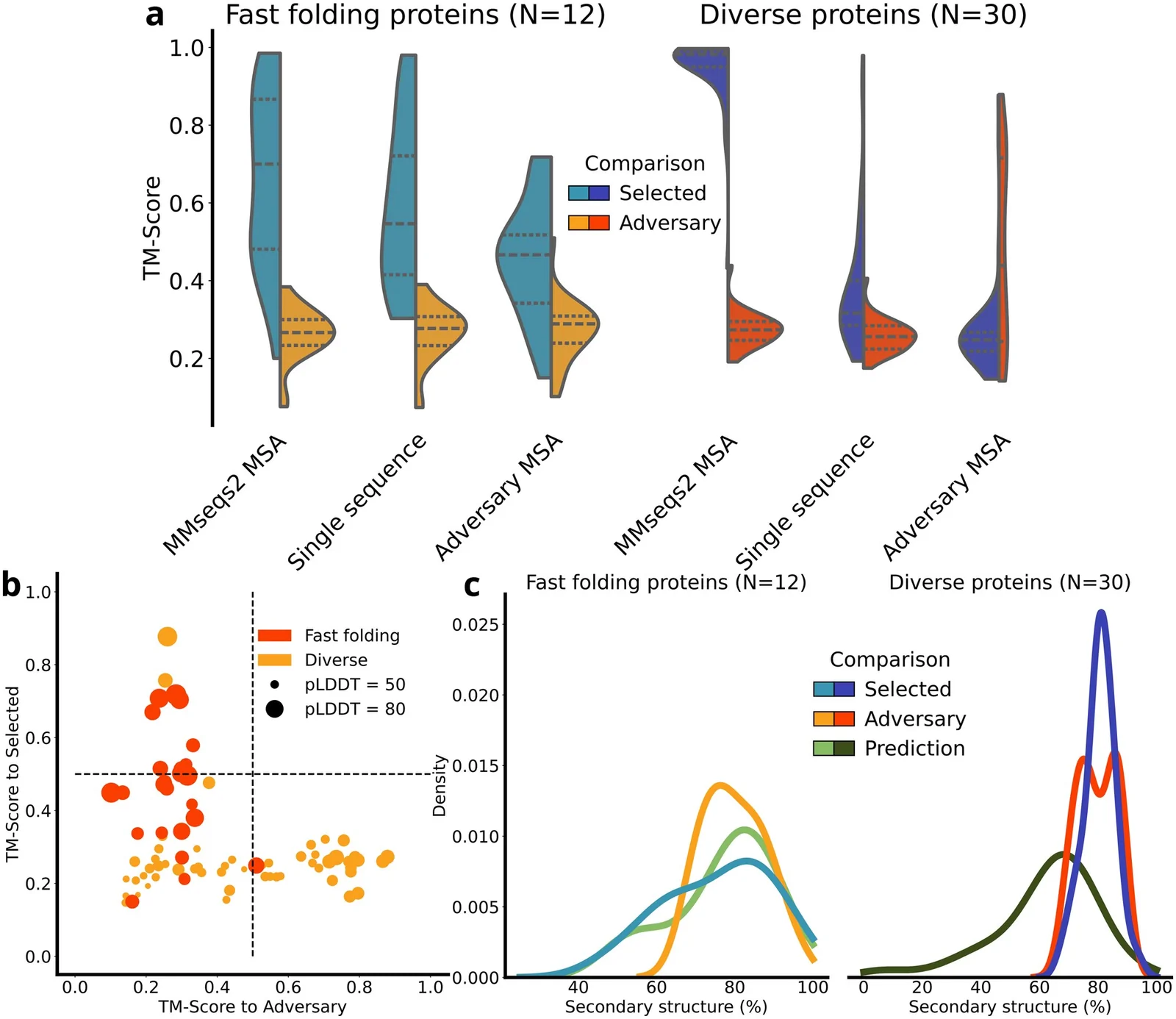
Impact of adversarial MSAs on structure prediction. (a) TM-score of different MSAs (MMseqs2, single sequence, synthetic MSA of adversary conformation) compared to selected PDB structure or adversary separated between *fast-folding* proteins and *diverse* proteins. (b) Scatter plot of TM-scores of *fast-folding* proteins and *diverse* proteins compared to selected conformation and adversary conformation with size of marker relative to pLDDT. (c) Secondary structure annotations of selected PDB, adversary conformation and the predicted structure with the adversary MSA separated between *fast-folding* proteins and *diverse* proteins.

We began by evaluating AlphaFold 2 predictions using standard MSAs obtained via the MMseqs2 pipeline. As expected, this baseline approach performed well across both datasets, with clear structural distinctions maintained between each *selected* protein and its paired *adversary* (Fig. 5a). Next, we assessed the effect of removing the MSA entirely. In this single-sequence setting, a clear difference emerged. Predictions for the *fast-folding* proteins retained a relatively high level of accuracy, whereas predictions for the structurally *diverse* proteins degraded considerably. The final and most informative condition used the *selected* protein’s sequence paired with a synthetic MSA designed to encode the structure of its *adversary*. For the *fast-folding* proteins, there is a noticeable decline in comparison to the MMseqs2 baseline; this decline is small, however, in comparison to the substantial decline observed for the *diverse* proteins. This suggests that the *fast-folding* proteins have a degree of robustness to MSA manipulation, which is supported by relatively consistent high pLDDT scores (Fig. 5b); thus again highlighting the limitation of the score for such conditions.

Notably, in approximately half of the *diverse* protein cases, AlphaFold 2 produced conformations resembling the *adversary* structure (TM-score > 0.6; Fig. 5b). In the other half, the predictions yielded alternative, partially folded conformations with an increased proportion of unfolded regions (Fig. 5c). Further analyses based on secondary structure (Joosten *et al.* 2011), contact order (Plaxco *et al.* 1998), and CATH classifications (Orengo *et al.* 1997) did not allow the identification of consistent features predictive of successful structural redirection (Table 12, available as supplementary data at *Bioinformatics* online).

To disentangle the influence of the adversarial MSA from the intrinsic properties of the input sequence, we conducted follow-up experiments on a subset of three mostly α-helical and three mostly β-sheet *selected* proteins that failed to adopt the adversarial fold in the original setup. For each of these targets, we paired the same input sequence with three different MSAs derived from trimmed *adversarial* proteins that managed to nudge the prediction in the previous setup. Despite this variation in MSA composition, none of the predictions successfully recapitulated the adversarial fold (Table 13, available as supplementary data at *Bioinformatics* online). This suggests that the failure to adopt alternative conformations is primarily determined by the intrinsic folding preferences of the input sequence, rather than by signals introduced through the adversarial MSAs.

### 3.4 Mapping AlphaFold 2’s distributional limits across the folding landscape

To probe the implicit folding landscape learned by AlphaFold2, we leveraged long-timescale molecular dynamics (MD) trajectories of fast-folding proteins (Lindorff-Larsen *et al.* 2011), which sample transitions between folded and partially unfolded states. Folded conformations would align with the training distribution of the model, while partially unfolded or high-entropy states would fall outside of it, enabling us to examine how the model resolves out-of-distribution structural inputs.

**Figure 6:**
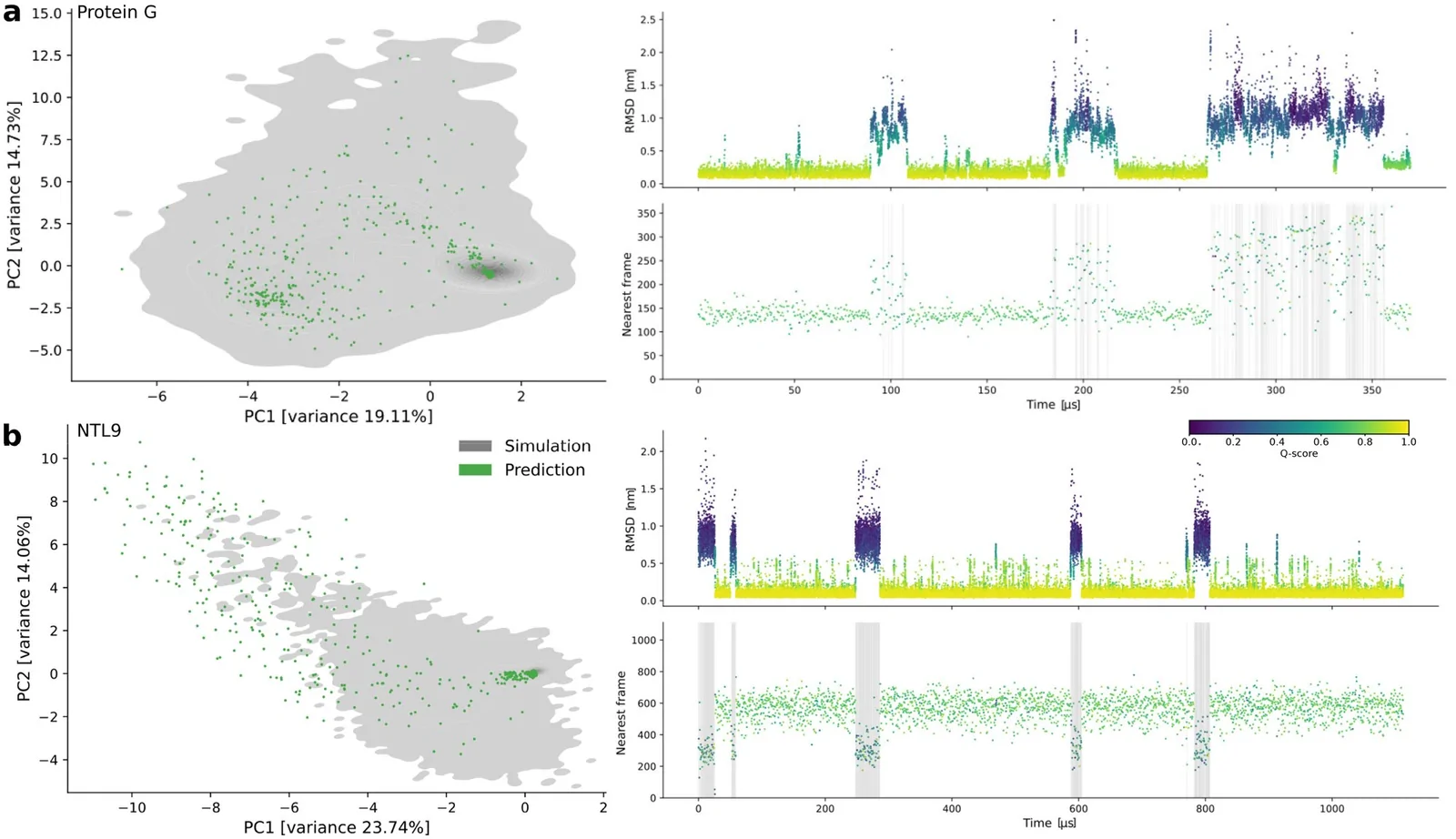
Conformational landscape from MD simulations of NTL9 (a) and Protein G (b) compared to AlphaFold 2 predictions. Projection of simulation frames onto the eigenspace defined by the first two principal components (left). Frame densities are represented as contour levels, with higher densities corresponding to more frequently sampled conformations. Green points indicate AlphaFold 2 predictions generated using frame-specific synthetic MSAs derived from the corresponding simulation frames. (Right) Timeline plots display simulation time on the *x*-axis, with RMSD to the experimental structure shown above and the index of the closest MD frames to each prediction shown below. To reduce noise, the average of the 20 nearest simulation frames is plotted. Predictions with fewer than five MD frames within 0.5 nm Cα RMSD are marked with vertical gray bars. Point coloring represents the Q-score Best *et al*. (2013) relative to the experimental structure.

We focused on three proteins, NTL9, Protein G variant NuG2, and Villin, selected based on their strong responses to adversarial MSAs in previous experiments. For each sampled MD frame, a synthetic MSA was generated using inverse folding to encode the frame-specific conformation while preserving the native sequence (examples can be seen in Fig. 5, available as supplementary data at *Bioinformatics* online). These frame-specific MSAs were supplied to AlphaFold 2 to assess whether the model could reconstruct the corresponding structural state. Structural read out was quantified by Cα RMSD relative to the MD trajectory, with predictions considered unresolved if fewer than five frames fell within 0.5 nm RMSD. We report the mean index of up to 20 closest matched frames for each protein (Fig. 6 and Fig. 4, available as supplementary data at *Bioinformatics* online).

Analysis of the predictions reveals a strong dependence on the conformational state sampled by MD. When MD snapshots are near the native fold, AlphaFold 2 reliably reads out the experimental structure. In contrast, snapshots corresponding to high-entropy states are often not reproduced. In most cases, the model predicts partially unfolded conformations that were not explicitly sampled in the MD trajectory but occupy regions within the principal component space defined by the simulation. Interestingly, in some cases, the native fold is read out even when using an MSA derived from an unfolded state.

To further characterize our observations, we performed high-temperature MD simulations of NTL9, Protein G, and Villin (Fig. 6, available as supplementary data at *Bioinformatics* online). Each simulation started from the experimentally determined folded structure and was run until the protein fully unfolded (details in Section 4.3). This setup produces a single, well-defined unfolding transition, avoiding multiple interconversions between folded and partially folded states. In this case, AlphaFold 2 also accurately reproduces the folded structure at the start of the trajectory, but as the protein unfolds and RMSD increases, its predictions increasingly fail to match the conformational space sampled by the MD simulation.

### 3.5 Hybrid MSAs allow targeted recovery of alternative conformations

Our results show that synthetic MSAs provide a powerful mechanism to direct protein structure predictors toward specific conformations. Since this strategy relies on structural information, we developed hybrid alignments (*FrankenMSAs*) that combine inverse-folded sequences for structurally known regions with MMseqs2 MSAs for other regions.

**Figure 7:**
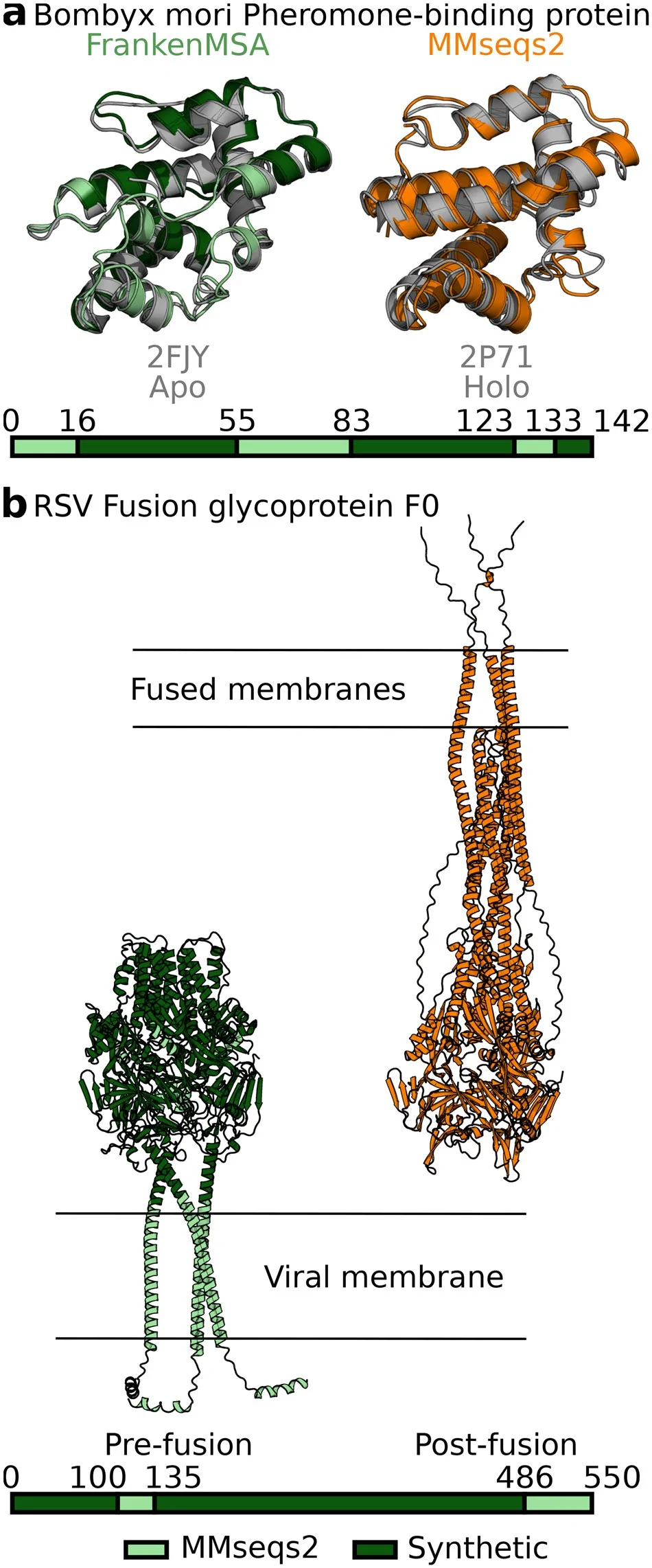
Comparison of AlphaFold structures using MMseqs2 MSA (orange structures) and FrankenMSA (green structures) using a combination of MMseqs2 and synthetic inverse folded parts indicated in the sequence bar with light green and deep green, respectively. (a) The *Bombyx mori* Pheromone-binding protein in the FrankenMSA-enforced *apo* conformation (2FJY) and the MMseqs2-preferred *holo* conformation (2P71). (b) The Respiratory syncytial virus (RSV) Fusion glycoprotein F0 in the FrankenMSA-enforced pre-fusion conformation (5U68) and the MMseqs2-preferred post-fusion conformation (3RRR). There are some MMseqs2 parts in the FrankenMSA structure of 5U68 to infer unmodeled domains.

As a first case study, we examined the monomeric B-form *Bombyx mori* Pheromone Binding Protein. Under default MMseqs2 AlphaFold 2 settings, the prediction corresponds to the *holo* conformation (PDB: 2P71) (Fig. 7a and Fig. 7c, available as supplementary data at *Bioinformatics* online). To bias predictions toward the *apo* state (2FJY), we inverse-folded sequence segments corresponding to the *apo* structural fragments and merged these synthetic segments with the remaining columns populated from the MMseqs2-derived MSA, producing a FrankenMSA that preserves the native sequence in the query row. Using this FrankenMSA as input to AlphaFold 2 successfully read out the *apo* fold with a TM-score of 0.916 to the experimental structure (Table 14, available as supplementary data at *Bioinformatics* online).

Next, we tested the method on an oligomeric system, the fusion (F) protein of Respiratory Syncytial Virus (RSV). Standard MMseqs2 AlphaFold 3 recapitulated the post-fusion conformation (Fig. 7b and Fig. 7, available as supplementary data at *Bioinformatics* online for trimers and monomers, respectively).To bias the prediction toward the pre-fusion conformation, we inverse-folded the core region associated with the pre-fusion fold (PDB: 5U68) and incorporated the resulting sequence columns into the protein’s MMseqs2 MSA. This FrankenMSA was then supplied to AlphaFold 3 for structure prediction. This procedure yielded models that matched the trimeric pre-fusion architecture observed experimentally (TM-score of 0.954 compared to pre-fusion reference), and additionally inferred previously unmodeled regions in the unfolded domain in the middle of the protein sequence and the transmembrane domain toward the C-terminal end. However, while each protomer adopted the correct pre-fusion fold, the relative packing of the three chains showed modest deviations from the experimental assembly (Tables 15 and 16, available as supplementary data at *Bioinformatics* online for trimer and monomer, respectively), indicating possible limitations of FrankenMSAs with regard to multimeric ensembles.

We therefore caution that FrankenMSA efficacy will depend on (i) which residue ranges are inverse-folded, (ii) the quality and depth of the retained natural MSA columns, and (iii) the protein structure prediction model used for structure modelling.

## 4 Discussion

Deep learning protein structure predictors encode rich representations of sequence and evolutionary information, yet the mechanisms by which these representations constrain predicted conformational outcomes remain poorly understood. To systematically interrogate these mechanisms, we generated synthetic MSAs via inverse folding, producing alignments that explicitly encode residue–residue correlations corresponding to specific conformations. By supplying these controlled inputs to AlphaFold2, AlphaFold3, and RoseTTAFold2, we manipulate what each model receives and directly observe how predictions respond, independently of the confounds introduced by natural sequence and evolutionary variation.

Conceptually, this setup can be understood as an encoder–decoder system (Bishop and Nasrabadi 2006): ProteinMPNN encodes structural information into sequences, which the structure predictor then decodes back into coordinates. Our results demonstrate that synthetic MSAs robustly influence model predictions across all three systems tested, extending previous observations from a smaller dataset (Wayment-Steele *et al.* 2025). Supplying alignments generated by inverse folding biases predicted structures toward target conformations even when the MSA consists entirely of sequences absent from sequence databases. This indicates that the models do not require evolutionary plausibility, but only internally consistent residue relationships, for an alignment to influence the predicted structure. The observation that removing sequences with detectable UniProt homologs caused only a modest drop in performance further supports this interpretation, suggesting that AlphaFold2 exploits residue co-variation patterns rather than simple sequence frequency statistics. These findings reveal that the information channel connecting MSA input to predicted conformation is sensitive to structural encoding, not merely evolutionary signal.

Adversarial experiments reveal a context-dependent integration of sequence and alignment information. For small, well-packed domains with strong intrinsic folding signals, predictions remain robust even when supplied with MSAs encoding a competing fold, indicating that sequence-derived constraints can dominate and suppress conflicting alignment information. Conversely, for proteins with weaker intrinsic sequence signals, misleading MSA information drives predictions toward unintended conformations or partially unfolded states. Follow-up experiments pairing the same input sequences with MSAs from multiple different adversarial proteins confirmed that resistance to redirection is primarily determined by the intrinsic folding preferences of the query sequence rather than by the specific MSA supplied. Importantly, pLDDT remained high across both outcomes, underscoring that this confidence metric reflects the internal consistency with which the model reconciles its inputs, rather than the biological accuracy or physiological relevance of the predicted structure. This has direct implications for how pLDDT should be interpreted when using engineered or non-natural MSAs.

Probing model behavior using synthetic MSAs derived from long-timescale molecular dynamics trajectories reveals a systematic dependence on conformational state. When trajectory frames are near the native fold, AlphaFold2 reliably reconstructs the experimental structure. As frames increasingly deviate toward partially unfolded or high-entropy states, predictions progressively fail to match the input conformation, instead relaxing toward alternative structural basins that are not explicitly present in the trajectory but fall within the principal component space of the simulation. This pattern, observed consistently across NTL9, Protein G, and Villin, is consistent with an inductive bias toward compact, training-distribution-favored conformations, reflecting the predominance of well-folded structures in the PDB. These observations align with the hypothesis that MSAs parametrize an implicit energy landscape learned by structure predictors (Roney and Ovchinnikov 2022), and suggest that synthetic MSAs can reshape this landscape sufficiently to shift the predicted minimum. We note, however, that this energy landscape interpretation is inferential rather than directly demonstrated, and the relationship between the implicit landscape encoded by these models and true thermodynamic stability remains an open question (Lewis *et al.* 2025, Outeiral *et al.* 2022). We also cannot exclude that some of these results reflect structural memorization of training-set folds (Lazou *et al.* 2024). However, consistent results on post-training-cutoff structures and adversarial experiments suggest this is not the primary driver. We note that the distinction between pattern recognition and generalization in the form of learned energetics, previously proposed as a framework for interpreting AF2 predictions (Chakravarty *et al.* 2024), may not fully capture the complexity of deep learning systems, in which memorization, generalization, and learned representations are not mutually exclusive (Goodfellow *et al.* 2016, Feldman 2020).

Synthetic and natural MSAs can be combined into hybrid alignments, which we term FrankenMSAs, to incorporate conformation-specific structural information for defined sequence regions while preserving evolutionary context elsewhere. Applied to the *Bombyx mori* Pheromone Binding Protein and the RSV Fusion glycoprotein, this strategy successfully steered predictions toward target conformations inaccessible under standard MMseqs2 inputs, and in the case of RSV additionally inferred previously unmodeled regions. These examples demonstrate that partial structural information can be integrated into MSA-based prediction workflows without requiring a complete structural template, extending the utility of synthetic MSAs to settings where only fragmentary structural data are available. FrankenMSA efficacy will depend on the selection of regions to be replaced by synthetic sequences, the quality of retained natural MSA columns, and the prediction model used; for oligomeric targets, coupling with inter-chain constraints, such as AlphaLink2 (Stahl *et al.* 2024) may further improve quaternary assembly control.

Several limitations of synthetic MSAs as a probing tool deserve consideration. First, generating synthetic MSAs requires a known or modelled target conformation, meaning the approach can only interrogate model responses to conformational states that are already structurally characterized. This restricts its use as an exploratory tool for discovering entirely unknown conformations. Second, while adversarial experiments reveal differential sensitivity to alignment-derived signals, the mechanistic basis for this variation remains unclear. We could not identify consistent sequence or structural features that reliably predict whether a model will follow or resist a synthetic MSA, limiting our ability to interpret these responses in terms of specific learned representations. Third, our experiments probe model behavior through input manipulation rather than direct inspection of internal representations, meaning the inferences we draw about learned conformational landscapes are necessarily indirect. More direct interpretability approaches, such as attention analysis or activation probing, could complement the external perturbation strategy introduced here. Fourth, synthetic MSAs are effective at directing predictions toward specific secondary structure compositions, but finer details, such as hinge angles and local conformational changes are harder to control. We attribute this to two factors: ProteinMPNN is trained with Gaussian noise on backbone positions, meaning minor structural variations are unlikely to be captured in the generated sequences; and subtle conformational differences may fall within the learned energy function of the prediction models, which allocate local geometry according to their own internal constraints regardless of MSA input.

Our findings establish that MSAs are not merely supporting input features but programmable levers for interrogating and manipulating structural outcomes. Synthetic MSAs offer a direct mechanism to encode conformation-specific information into structure predictors, effectively expanding the ensemble of accessible states and exposing the boundaries of learned conformational space. We anticipate that treating MSAs as a design space (Rao *et al.* 2021), analogous to sequence or structure, will open new strategies for conformation control, model interrogation, and systematic data augmentation. As generative models for MSAs continue to advance (Sturmfels *et al.* 2022, Zhang *et al.* 2023, 2024, Chen *et al.* 2024), bridging alignment design with structure prediction will become increasingly central to building more interpretable and controllable modelling workflows.

## Supplementary Material

btag605_Supplementary_Data

## Data Availability

Implementation code and changes to the fold-switching dataset Porter and Looger (2018) are available on GitHub: https://github.com/ibmm-unibe-ch/msa-tests our generated data from predictions is available on 10.5281/zenodo.20916910. We make a tool available to easily use inverse folded MSAs: https://colab.research.google.com/github/ibmm-unibe-ch/msa-tests/blob/master/FrankenMSA.ipynb.
