# Supplementary material for: Synthetic sequence alignments as programmable probes of learned conformational landscapes in deep learning protein structure predictors
Source: Bioinformatics. 2026 Aug 11;42(8):btag605. doi: 10.1093/bioinformatics/btag605 (PMC13504272; doi:10.1093/bioinformatics/btag605)
Supplement: Supplementary file 1 [file btag605_supplementary_data.pdf]

Supporting information for ”Synthetic sequence alignments as programmable probes of learned conformational landscapes in deep learning protein structure predictors”

<table>
<tr><th>ID (A)</th><th>ID (B)</th><th>Year (A)</th><th>Year (B)</th><th>Length (A)</th><th>Length (B)</th><th>Type</th><th>Offset (A)</th><th>Offset (B)</th></tr>
<tr><td>7ahl|E</td><td>4yhd|G</td><td>1998</td><td>2015</td><td>53</td><td>53</td><td>C</td><td>100</td><td>100</td></tr>
<tr><td>1rep|C</td><td>2z9o|B</td><td>2000</td><td>2007</td><td>17</td><td>17</td><td>D</td><td>139</td><td>154</td></tr>
<tr><td>3tp2|A</td><td>5lj3|M</td><td>2012</td><td>2016</td><td>42</td><td>42</td><td>A</td><td>5</td><td>3</td></tr>
<tr><td>1g2c|F</td><td>5c6b|F</td><td>2011</td><td>2015</td><td>36</td><td>38</td><td>B</td><td>3</td><td>429</td></tr>
<tr><td>5ec5|P</td><td>3zxg|B</td><td>2016</td><td>2012</td><td>54</td><td>54</td><td>C</td><td>45</td><td>43</td></tr>
<tr><td>1uxm|K</td><td>2nam|A</td><td>2009</td><td>2015</td><td>153</td><td>153</td><td>C</td><td>0</td><td>8</td></tr>
<tr><td>5aoe|B</td><td>5ly6|B</td><td>2017</td><td>2017</td><td>128</td><td>128</td><td>D</td><td>172</td><td>155</td></tr>
<tr><td>1ova|A</td><td>1jti|B</td><td>1992</td><td>2001</td><td>52</td><td>52</td><td>D</td><td>311</td><td>311</td></tr>
<tr><td>3gmh|L</td><td>2vfx|L</td><td>2010</td><td>2002</td><td>38</td><td>38</td><td>D</td><td>166</td><td>165</td></tr>
<tr><td>3m1b|F</td><td>3low|A</td><td>1998</td><td>2010</td><td>21</td><td>21</td><td>C</td><td>40</td><td>41</td></tr>
<tr><td>3j7w|B</td><td>3j7v|G</td><td>2014</td><td>2014</td><td>21</td><td>21</td><td>C</td><td>22</td><td>22</td></tr>
<tr><td>2lqw|A</td><td>2bzy|B</td><td>2012</td><td>2006</td><td>67</td><td>67</td><td>C</td><td>236</td><td>0</td></tr>
<tr><td>5hmg|B</td><td>1htm|B</td><td>1990</td><td>1995</td><td>113</td><td>113</td><td>B</td><td>58</td><td>21</td></tr>
<tr><td>4j3o|F</td><td>2jmr|A</td><td>2013</td><td>2007</td><td>25</td><td>25</td><td>C</td><td>0</td><td>0</td></tr>
<tr><td>1miq|B</td><td>1qs8|B</td><td>2002</td><td>1999</td><td>32</td><td>32</td><td>D</td><td>46</td><td>0</td></tr>
<tr><td>4nc9|C</td><td>4n9w|A</td><td>2015</td><td>2015</td><td>21</td><td>21</td><td>D</td><td>121</td><td>121</td></tr>
<tr><td>3j97|M</td><td>1xtg|B</td><td>2015</td><td>2004</td><td>40</td><td>40</td><td>A</td><td>156</td><td>17</td></tr>
<tr><td>2nao|F</td><td>1iyt|A</td><td>2016</td><td>2002</td><td>28</td><td>28</td><td>C</td><td>14</td><td>14</td></tr>
<tr><td>5c1v|A</td><td>5c1v|B</td><td>2016</td><td>2016</td><td>31</td><td>31</td><td>D</td><td>303</td><td>303</td></tr>
<tr><td>5jyt|A</td><td>2qke|E</td><td>2013</td><td>2017</td><td>53</td><td>54</td><td>A</td><td>50</td><td>50</td></tr>
<tr><td>2lej|A</td><td>2lv1|A</td><td>2011</td><td>2012</td><td>15</td><td>15</td><td>C</td><td>28</td><td>28</td></tr>
<tr><td>3jv6|A</td><td>1zk9|A</td><td>2010</td><td>2005</td><td>29</td><td>29</td><td>A</td><td>38</td><td>47</td></tr>
<tr><td>5fhc|J</td><td>1ebo|E</td><td>2016</td><td>1999</td><td>56</td><td>56</td><td>B</td><td>42</td><td>24</td></tr>
<tr><td>4wsg|C</td><td>1svf|C</td><td>2015</td><td>1999</td><td>39</td><td>39</td><td>B</td><td>124</td><td>24</td></tr>
<tr><td>1qom|B</td><td>1noc|A</td><td>1999</td><td>1997</td><td>22</td><td>22</td><td>C</td><td>393</td><td>343</td></tr>
<tr><td>5keq|F</td><td>1dzl|A</td><td>2017</td><td>2000</td><td>34</td><td>34</td><td>C</td><td>407</td><td>409</td></tr>
<tr><td>4y0m|J</td><td>4xws|D</td><td>2015</td><td>2015</td><td>25</td><td>25</td><td>D</td><td>136</td><td>136</td></tr>
<tr><td>2c1u|C</td><td>2c1v|B</td><td>2006</td><td>2006</td><td>35</td><td>35</td><td>D</td><td>221</td><td>221</td></tr>
<tr><td>4qhf|A</td><td>4qhh|A</td><td>2014</td><td>2014</td><td>19</td><td>19</td><td>C</td><td>75</td><td>75</td></tr>
<tr><td>4aan|A</td><td>4aal|A</td><td>2012</td><td>2012</td><td>34</td><td>34</td><td>D</td><td>226</td><td>226</td></tr>
<tr><td>1x0g|A</td><td>1x0g|D</td><td>2006</td><td>2006</td><td>70</td><td>68</td><td>C</td><td>42</td><td>42</td></tr>
<tr><td>4ae0|A</td><td>4ow6|B</td><td>2012</td><td>2014</td><td>12</td><td>12</td><td>D</td><td>258</td><td>258</td></tr>
<tr><td>3ifa|A</td><td>5et5|A</td><td>2010</td><td>2016</td><td>12</td><td>12</td><td>D</td><td>8</td><td>8</td></tr>
<tr><td>1h38|D</td><td>1qln|A</td><td>2002</td><td>2000</td><td>16</td><td>16</td><td>D</td><td>588</td><td>588</td></tr>
<tr><td>1xjt|A</td><td>1xju|B</td><td>2005</td><td>2005</td><td>30</td><td>31</td><td>D</td><td>38</td><td>10</td></tr>
<tr><td>3hde|A</td><td>3hdf|A</td><td>2009</td><td>2009</td><td>12</td><td>12</td><td>D</td><td>133</td><td>108</td></tr>
<tr><td>3ejh|A</td><td>3m7p|A</td><td>2009</td><td>2010</td><td>23</td><td>23</td><td>A</td><td>0</td><td>219</td></tr>
<tr><td>1k0n|A</td><td>1rk4|B</td><td>2001</td><td>2003</td><td>42</td><td>42</td><td>B</td><td>36</td><td>38</td></tr>
<tr><td>1xnt|A</td><td>3lqc|A</td><td>2010</td><td>1999</td><td>23</td><td>23</td><td>D</td><td>16</td><td>16</td></tr>
<tr><td>4gqc|C</td><td>4gqc|B</td><td>2012</td><td>2012</td><td>23</td><td>23</td><td>D</td><td>45</td><td>45</td></tr>
<tr><td>4dxt|A</td><td>4dxr|A</td><td>2012</td><td>2012</td><td>30</td><td>30</td><td>C</td><td>38</td><td>42</td></tr>
<tr><td>4rwn|A</td><td>4rwq|B</td><td>2015</td><td>2015</td><td>14</td><td>14</td><td>A</td><td>60</td><td>60</td></tr>
<tr><td>2hdm|A</td><td>2n54|B</td><td>2001</td><td>2008</td><td>17</td><td>17</td><td>B</td><td>48</td><td>49</td></tr>
<tr><td>3vo9|B</td><td>3vpa|D</td><td>2012</td><td>2012</td><td>14</td><td>14</td><td>D</td><td>184</td><td>184</td></tr>
<tr><td>2p3v|A</td><td>2p3v|D</td><td>2007</td><td>2007</td><td>16</td><td>16</td><td>D</td><td>23</td><td>23</td></tr>
<tr><td>3ews|B</td><td>3g0h|A</td><td>2009</td><td>2009</td><td>10</td><td>10</td><td>D</td><td>43</td><td>22</td></tr>
<tr><td>2ce7|C</td><td>3kds|G</td><td>2006</td><td>2009</td><td>17</td><td>17</td><td>D</td><td>301</td><td>301</td></tr>
<tr><td>4phq|A</td><td>2wcd|X</td><td>2014</td><td>2009</td><td>17</td><td>17</td><td>B</td><td>173</td><td>184</td></tr>
<tr><td>3t1p|A</td><td>1kct|A</td><td>2011</td><td>1996</td><td>84</td><td>84</td><td>D</td><td>257</td><td>280</td></tr>
<tr><td>5l35|D</td><td>5l35|G</td><td>2017</td><td>2017</td><td>28</td><td>28</td><td>D</td><td>178</td><td>178</td></tr>
<tr><td>5f3k|A</td><td>5f5r|B</td><td>2016</td><td>2016</td><td>25</td><td>25</td><td>B</td><td>26</td><td>26</td></tr>
<tr><td>4qds|A</td><td>2qqj|A</td><td>2015</td><td>2007</td><td>20</td><td>20</td><td>B</td><td>163</td><td>162</td></tr>
<tr><td>5jzh|A</td><td>5jzt|G</td><td>2016</td><td>2016</td><td>64</td><td>64</td><td>C</td><td>214</td><td>214</td></tr>
<tr><td>4pyi|A</td><td>4pyj|A</td><td>2014</td><td>2014</td><td>14</td><td>14</td><td>C</td><td>202</td><td>202</td></tr>
<tr><td>5ine|A</td><td>3mko|A</td><td>2016</td><td>2010</td><td>21</td><td>21</td><td>B</td><td>358</td><td>58</td></tr>
<tr><td>2a73|B</td><td>3l5n|B</td><td>2005</td><td>2005</td><td>37</td><td>37</td><td>D</td><td>813</td><td>735</td></tr>
<tr><td>2k0q|A</td><td>2lel|A</td><td>2011</td><td>2008</td><td>23</td><td>23</td><td>D</td><td>48</td><td>48</td></tr>
<tr><td>3uyi|A</td><td>3v0t|A</td><td>2012</td><td>2012</td><td>15</td><td>15</td><td>B</td><td>205</td><td>204</td></tr>
<tr><td>4a5w|B</td><td>3t5o|A</td><td>2012</td><td>2012</td><td>16</td><td>16</td><td>A</td><td>223</td><td>223</td></tr>
<tr><td>1rkp|A</td><td>2h44|A</td><td>2004</td><td>2006</td><td>22</td><td>22</td><td>D</td><td>121</td><td>121</td></tr>
<tr><td>1cee|B</td><td>2k42|A</td><td>1999</td><td>2008</td><td>24</td><td>20</td><td>A</td><td>8</td><td>4</td></tr>
<tr><td>3o44|A</td><td>1xez|A</td><td>2011</td><td>2005</td><td>18</td><td>18</td><td>C</td><td>169</td><td>297</td></tr>
<tr><td>3kuy|A</td><td>5c3i|F</td><td>2009</td><td>2015</td><td>16</td><td>16</td><td>C</td><td>46</td><td>47</td></tr>
<tr><td>2n0a|D</td><td>2kkw|A</td><td>2004</td><td>2015</td><td>138</td><td>138</td><td>C</td><td>0</td><td>0</td></tr>
<tr><td>4m4r|A</td><td>4w50|B</td><td>2013</td><td>2014</td><td>14</td><td>14</td><td>D</td><td>127</td><td>127</td></tr>
<tr><td>5ejb|C</td><td>1wp8|C</td><td>2016</td><td>2005</td><td>38</td><td>39</td><td>B</td><td>116</td><td>4</td></tr>
<tr><td>1mby|A</td><td>4yyp|A</td><td>2002</td><td>2015</td><td>16</td><td>15</td><td>C</td><td>70</td><td>69</td></tr>
<tr><td>2nxq|B</td><td>1jfk|A</td><td>2007</td><td>2001</td><td>19</td><td>19</td><td>B</td><td>46</td><td>46</td></tr>
<tr><td>3j9c|A</td><td>3q8f|A</td><td>2015</td><td>2012</td><td>12</td><td>12</td><td>C</td><td>171</td><td>344</td></tr>
<tr><td>4rmb|A</td><td>4rmb|B</td><td>2015</td><td>2015</td><td>15</td><td>15</td><td>C</td><td>143</td><td>143</td></tr>
<tr><td>3njq|A</td><td>2pbk|B</td><td>2017</td><td>2007</td><td>25</td><td>23</td><td>D</td><td>6</td><td>5</td></tr>
<tr><td>3zwg|N</td><td>4tsy|D</td><td>2015</td><td>2015</td><td>12</td><td>12</td><td>B</td><td>3</td><td>3</td></tr>
<tr><td>4hdd|A</td><td>2lep|A</td><td>2013</td><td>2012</td><td>25</td><td>25</td><td>B</td><td>16</td><td>16</td></tr>
<tr><td>5k5g|A</td><td>2kb8|A</td><td>2016</td><td>2009</td><td>21</td><td>21</td><td>C</td><td>9</td><td>9</td></tr>
<tr><td>1nqd|A</td><td>1nqj|B</td><td>2003</td><td>2003</td><td>33</td><td>31</td><td>D</td><td>0</td><td>0</td></tr>
<tr><td>5flu|E</td><td>2uy7|D</td><td>2016</td><td>2007</td><td>23</td><td>23</td><td>C</td><td>7</td><td>7</td></tr>
<tr><td>2oug|C</td><td>2lcl|A</td><td>2012</td><td>2007</td><td>41</td><td>47</td><td>A</td><td>115</td><td>16</td></tr>
<tr><td>4zrb|C</td><td>4zrb|H</td><td>2015</td><td>2015</td><td>20</td><td>20</td><td>D</td><td>4</td><td>4</td></tr>
<tr><td>1mnm|C</td><td>1mnm|D</td><td>1998</td><td>1998</td><td>13</td><td>13</td><td>C</td><td>13</td><td>13</td></tr>
<tr><td>2nnt|A</td><td>2mwf|A</td><td>2015</td><td>2006</td><td>25</td><td>25</td><td>C</td><td>3</td><td>0</td></tr>
<tr><td>4fu4|C</td><td>4g0d|Z</td><td>2013</td><td>2013</td><td>23</td><td>23</td><td>D</td><td>3</td><td>3</td></tr>
<tr><td>4b3o|B</td><td>3mee|A</td><td>2013</td><td>2010</td><td>40</td><td>40</td><td>D</td><td>401</td><td>400</td></tr>
<tr><td>4twa|A</td><td>4ydq|B</td><td>2014</td><td>2015</td><td>38</td><td>38</td><td>D</td><td>80</td><td>80</td></tr>
<tr><td>2axz|A</td><td>2grm|B</td><td>2005</td><td>2005</td><td>14</td><td>15</td><td>D</td><td>292</td><td>292</td></tr>
<tr><td>4o0p|A</td><td>4o01|D</td><td>2014</td><td>2014</td><td>13</td><td>13</td><td>D</td><td>479</td><td>479</td></tr>
<tr><td>4rr2|D</td><td>3l9q|B</td><td>2015</td><td>2010</td><td>16</td><td>16</td><td>A</td><td>315</td><td>46</td></tr>
<tr><td>5i2m|A</td><td>5i2s|A</td><td>2006</td><td>2007</td><td>27</td><td>27</td><td>B</td><td>382</td><td>382</td></tr>
</table>

Table S1: All inspected conformation pairs for the analysis in subsection 2.1. Both conformers (A,B) are noted by ID, which consists of the PDB ID and the chain identifier, followed by year of deposition, the length of the inspected conformation and the offset starting from the start of the adjacent input sequence. The types of the conformations are also noted according to the original publication [10].

<table>
<tr><th>ID (A)</th><th>ID (B)</th><th>Year (A)</th><th>Year (B)</th><th>Length (A)</th><th>Length (B)</th><th>Type</th><th>Offset (A)</th><th>Offset (B)</th><th>Change</th></tr>
<tr><td>5b3z|A</td><td>5bmy|A</td><td>2016</td><td>2016</td><td>29</td><td>11</td><td>D</td><td>7</td><td>7</td><td>Discarded because vastly different length</td></tr>
<tr><td>1wyy|B</td><td>5wrg|A</td><td>2005</td><td>2017</td><td>58</td><td>216</td><td>B</td><td>90</td><td>972</td><td>Discarded because vastly different length</td></tr>
<tr><td>2frh|A</td><td>1fzp|D</td><td>2006</td><td>2001</td><td>92</td><td>92</td><td>A</td><td>31</td><td>27</td><td>Discarded because obsoleted structure 1fzp|D</td></tr>
<tr><td>3k2s|A</td><td>2a01|C</td><td>2010</td><td>2006</td><td>39</td><td>39</td><td>D</td><td>138</td><td>138</td><td>Discarded because obsoleted structure 2a01|C</td></tr>
<tr><td>2ged|B</td><td>1nrj|B</td><td>2006</td><td>2003</td><td>20</td><td>20</td><td>B</td><td>76</td><td>40</td><td>Discarded because 2ged|B is only defined for 10/20 residues</td></tr>
<tr><td>4uv2|D</td><td>4q79|F</td><td>2014</td><td>2014</td><td>28</td><td>29</td><td>C</td><td>340</td><td>248</td><td>Discarded because 4q79|F is only defined for 4 residues of region of interest</td></tr>
<tr><td>3qy2|A</td><td>1qb3|A</td><td>2011</td><td>2000</td><td>15</td><td>15</td><td>C</td><td>102</td><td>102</td><td>Discarded because 3qy2|A is only defined until 105 making the overlap minimal</td></tr>
<tr><td>4zt0|C</td><td>4cmq|B</td><td>2015</td><td>2014</td><td>168</td><td>168</td><td>D</td><td>442</td><td>445</td><td>Discarded because half of this region is unmodeled in 4cmqB.</td></tr>
<tr><td>4jph|B</td><td>5hk5|H</td><td>2013</td><td>2016</td><td>22</td><td>22</td><td>A</td><td>25</td><td>23</td><td>Discarded because 4jph is only defined for an overlap of 7 residues in chain B.</td></tr>
<tr><td>4rr2|D</td><td>3l9q|B</td><td>2015</td><td>2010</td><td>46</td><td>46</td><td>A</td><td>315</td><td>46</td><td>Cut because 19 out of 46 residues missing in 3l9q|B PDB, only use first 16 residues instead</td></tr>
<tr><td>2nxq|B</td><td>1jfk|A</td><td>2007</td><td>2001</td><td>87</td><td>87</td><td>B</td><td>46</td><td>46</td><td>Cut because 2nxq|B doesn't have all indicated amino acids in pdb, only use overlapping 19 instead</td></tr>
<tr><td>3ews|B</td><td>3g0h|A</td><td>2009</td><td>2009</td><td>12</td><td>12</td><td>D</td><td>43</td><td>22</td><td>Cut because last two residues are not in the PDB of 3ews|B</td></tr>
<tr><td>3j9c|A</td><td>3q8f|A</td><td>2015</td><td>2012</td><td>22</td><td>22</td><td>C</td><td>161</td><td>334</td><td>Cut because first half of 3q8f|A has many missing residues in PDB, shorten region of interest</td></tr>
<tr><td>5k5g|A</td><td>2kb8|A</td><td>2016</td><td>2009</td><td>28</td><td>28</td><td>C</td><td>9</td><td>9</td><td>Cut because 5k5g|A is not defined for the whole 28 residues, only use 21</td></tr>
<tr><td>2hdm|A</td><td>2n54|B</td><td>2001</td><td>2008</td><td>22</td><td>22</td><td>B</td><td>48</td><td>49</td><td>Cut because 2n54|B is not defined for the whole 22 residues, only take 17 residues instead</td></tr>
<tr><td>2nnt|A</td><td>2mwf|A</td><td>2015</td><td>2006</td><td>37</td><td>32</td><td>C</td><td>3</td><td>0</td><td>Cut because 2nnt|A is only defined over 25 of the 37 residues, adapt accordingly</td></tr>
<tr><td>2axz|A</td><td>2grm|B</td><td>2005</td><td>2005</td><td>21</td><td>22</td><td>D</td><td>292</td><td>292</td><td>Cut because 2axz|A is only defined for the first 14 residues; can't run MD, because 2axz|A has a m</td></tr>
<tr><td>5flu|E</td><td>2uy7|D</td><td>2016</td><td>2007</td><td>30</td><td>30</td><td>C</td><td>0</td><td>0</td><td>Cut because 2uy7|D is only defined after 7 residues</td></tr>
<tr><td>4rmb|A</td><td>4rmb|B</td><td>2015</td><td>2015</td><td>17</td><td>17</td><td>C</td><td>143</td><td>143</td><td>Cut because 4rmb|A is not defined for the last 2 residues, needs to be shortened to 15</td></tr>
<tr><td>1rep|C</td><td>2z9o|A</td><td>2000</td><td>2007</td><td>17</td><td>17</td><td>D</td><td>139</td><td>154</td><td>Changed 2z9o|D to 2z9o|B</td></tr>
<tr><td>5i2m|A</td><td>5i2s|A</td><td>2006</td><td>2007</td><td>40</td><td>40</td><td>B</td><td>382</td><td>382</td><td>Cut because first 13 are not modeled by 5i2m|A</td></tr>
<tr><td>2oug|C</td><td>2lcl|A</td><td>2012</td><td>2007</td><td>61</td><td>65</td><td>A</td><td>100</td><td>0</td><td>Cut because first 15 as 2oug|C is not defined at the start and at the end.</td></tr>
<tr><td>5ine|A</td><td>3mko|A</td><td>2016</td><td>2010</td><td>48</td><td>48</td><td>B</td><td>358</td><td>58</td><td>Cut because 3mko|A not defined in middle, only take first part</td></tr>
<tr><td>4nc9|C</td><td>4n9w|A</td><td>2015</td><td>2015</td><td>30</td><td>30</td><td>D</td><td>121</td><td>121</td><td>Cut because last 9 residues because not defined in 4nc9|C.</td></tr>
<tr><td>3zwg|N</td><td>4tsy|D</td><td>2015</td><td>2015</td><td>15</td><td>15</td><td>B</td><td>0</td><td>0</td><td>Cut because 4tsy|D is only defined starting at 3</td></tr>
<tr><td>4y0m|J</td><td>4xws|D</td><td>2015</td><td>2015</td><td>69</td><td>69</td><td>D</td><td>92</td><td>92</td><td>Cut because 4xws|D has a long unmodeled part before 136.</td></tr>
</table>

Table S2: All changes to the original conformation dataset. Both conformers (A,B) are noted by ID, which consists of the PDB ID and the chain identifier, followed by year of deposition, the length of the inspected conformation and the offset starting from the start of the adjacent input sequence. The types of the conformations are also noted according to the original publication [10] as well as the committed change.

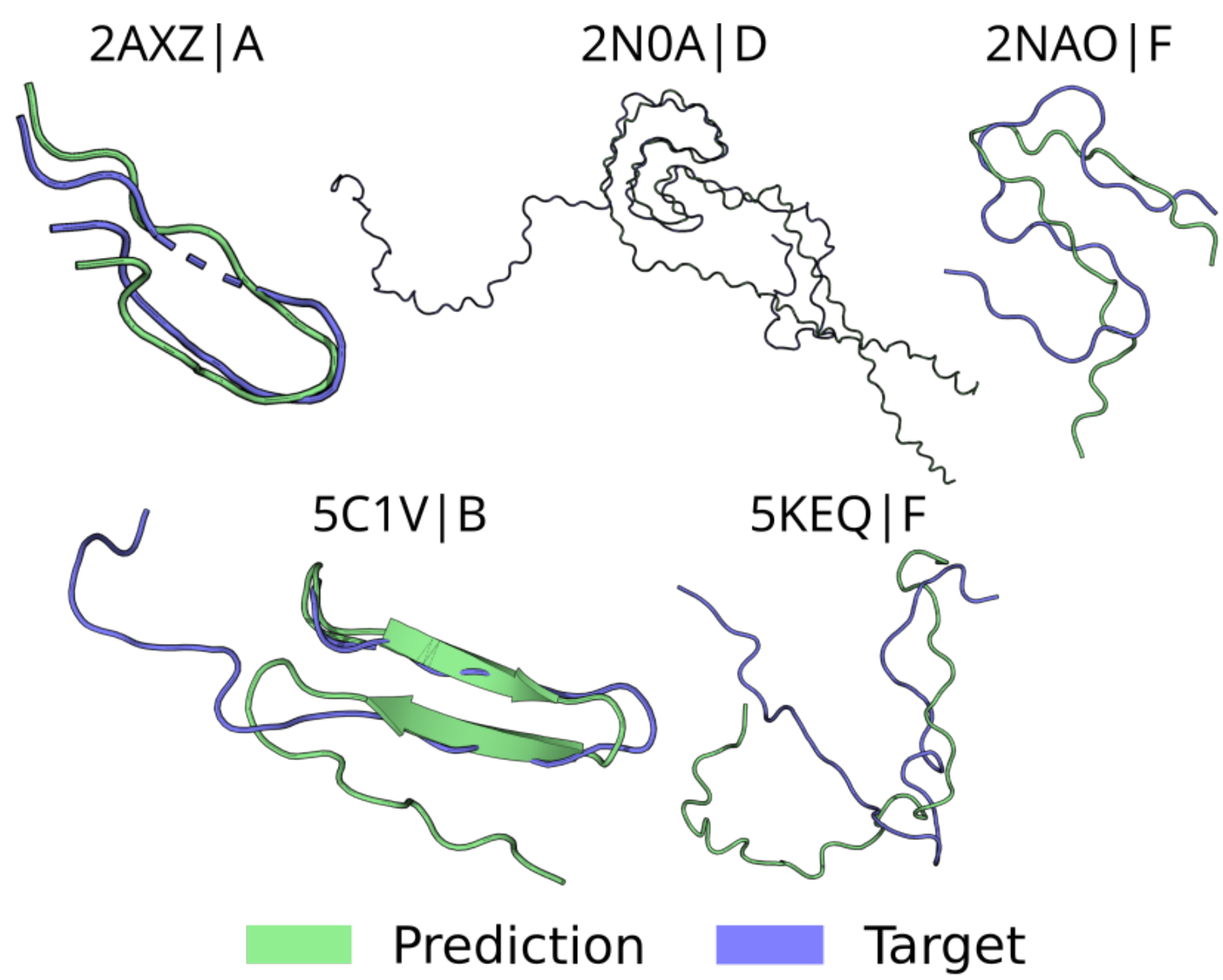

Figure S1: The five unfolded proteins (blue) and the predictions (green) of the conformation dataset.

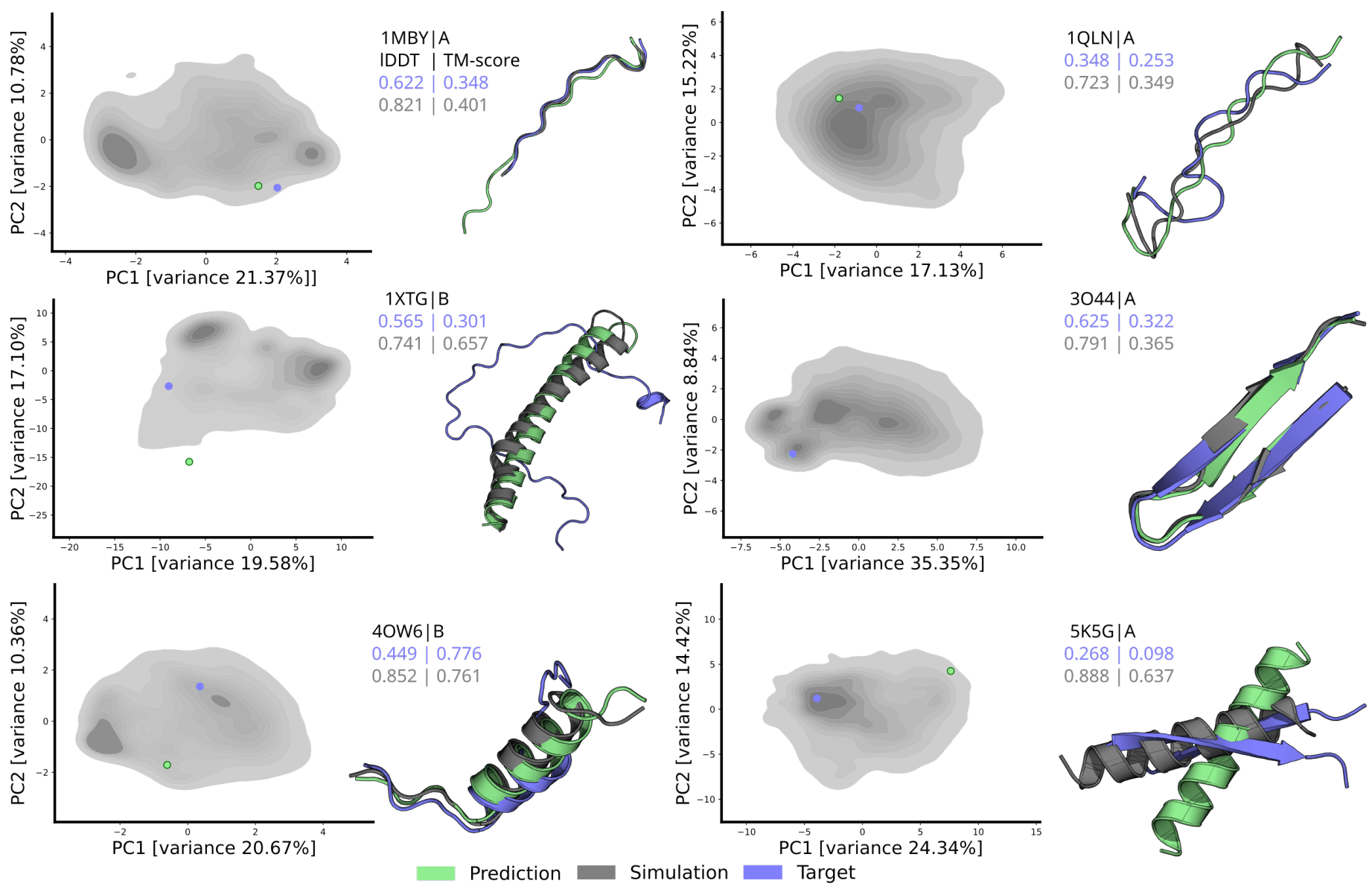

Figure S2: Additional representative structures obtained from molecular dynamics simulations with default parameters. PCA plots (left) depict the conformational space sampled during the MD simulations, projected onto the first two eigenvectors. lDDT and TM-scores comparing the prediction from the synthetic MSA to the initial target (blue) and to the closest simulation frame (gray) are reported. Cartoon representations show the closest simulation frame (gray), the target structure (blue), and the prediction (green). PCA Area around target is sometimes too shallow to be depicted in visualisation.

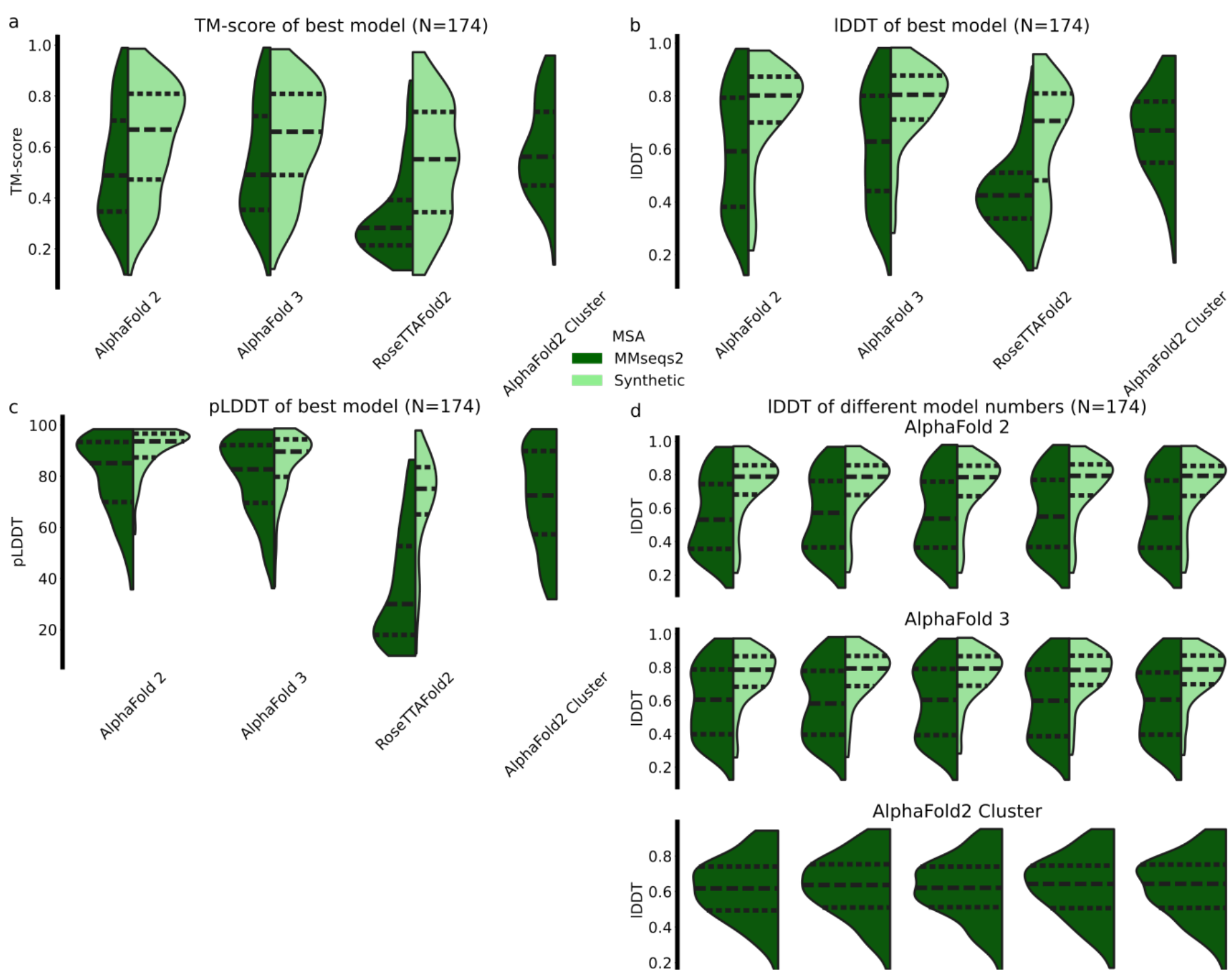

Figure S3: Additional ablation graphs. **a** TM-score of best model of four different protein structure prediction models. **b** lDDT of best model of four different protein structure prediction models. **c** pLDDT of best model of four different protein structure prediction models. **d** lDDT of every model of AlphaFold 2 AlphaFold 3 and AlphaFold 2 Cluster.

Metric	Model	Unpaired t-test		Paired t-test	
		t	p	t	p
TM-score	AlphaFold 2	4.514	4.36×10^{-6}	7.134	1.28×10^{-11}
	AlphaFold 3	4.418	6.67×10^{-6}	7.073	1.80×10^{-11}
	RoseTTAFold2	9.941	5.97×10^{-21}	11.482	2.40×10^{-23}
lDDT	AlphaFold 2	7.120	3.13×10^{-12}	10.789	2.21×10^{-21}
	AlphaFold 3	8.079	5.50×10^{-15}	10.663	5.04×10^{-21}
	RoseTTAFold2	10.859	3.90×10^{-24}	14.434	8.29×10^{-32}

Table S3: Comparison of TM-score and lDDT using unpaired and paired one tailed t-tests of the fold-switching dataset (N=174) comparing MMseqs2 and synthetic MSA predictions.

Model	MSA	Structure
AlphaFold 2 Cluster	MMseqs2	2LV1|A
AlphaFold 2 Cluster	MMseqs2	1NQD|A
AlphaFold 3	MMseqs2	4YDQ|B
AlphaFold 3	Synthetic	5C3I|F
AlphaFold 3	Synthetic	2LEJ|A
AlphaFold 3	Synthetic	4AAL|A
AlphaFold 3	Synthetic	1MNM|C
AlphaFold 3	Synthetic	4PHQ|A
AlphaFold 3	Synthetic	3NJQ|A
AlphaFold 3	Synthetic	1XJU|B
AlphaFold 2	Synthetic	2A73|B

Table S4: Resolution successes that have only been achieved by a single model, MSA combination.

Metric	MMseqs2	Synthetic
Entropy	1.483	2.003
Occupancy	0.682	1.000
Meff 25	1.607	22.927
Meff 50	285.776	128.880
Meff 80	3252.083	129.000
Identity	0.302	0.272
BLOSUM62 score	-0.108	-0.496
Type frequency	0.445	0.486
Type consensus frequency	0.633	0.583
Type entropy	1.295	1.406

Table S5: Metrics to analyse MSAs generated for the fold switching dataset (N=174) of MMseqs2 without insertions and the synthetic pipeline. We compute each metric per protein and then compute the average. We show Shannon entropy, occupancy (positions without gaps), Meff [7] at three different sequence identity cut-offs, sequence identity, BLOSUM62 score [5], type frequency of query sequence residue, type frequency of consensus type and type entropy with four standard types (acidic, basic, uncharged polar, nonpolar) [1]. Additionally, we computed the Spearman correlation [11] of residue entropies within a protein between MMseqs2 and synthetic. Across all proteins, the average is 0.294 or 0.303 after debiasing [3].

TM-score	2FS1	8E6Y
2FS1	1.000	0.450
MMseqs2	0.482	0.616
Synthetic (2FS1)	0.803	0.481
Synthetic (8E6Y)	0.484	0.659

Table S6: TM-scores of the SA1 test case [2]. The AlphaFold2 experiments were computed on the full 8E6Y|A and 9A20|A, which is a homologue to 2FS1|A. To accommodate some comparison to previous pipelines and disregard disordered regions, we measure on the domains modelled by 2FS1|A.

TM-score	6RVX	7BCQ
6RVX	1.000	0.628
MMseqs2	0.626	0.931
Synthetic (6RVX)	0.951	0.628
Synthetic (7BCQ)	0.629	0.956

Table S7: TM-scores of the ASCT2 test case [4]. The AlphaFold2 experiments were computed on 6RVX|A residues 4-117 and 7BCQ|A residues 1-113 due to unmodeled domains. We selected the domain with the biggest TM-score difference.

TM-score	7AAR	7AAQ
7AAR	1.000	0.816
MMseqs2	0.877	0.949
Synthetic (7AAR)	0.992	0.828
Synthetic (7AAQ)	0.819	0.993

Table S8: TM-scores of the STP10 test case [4]. The AlphaFold2 experiments were computed on the full 7AAR|A and 7AAQ|A.

TM-score	6XPF	6XPD
6XPF	1.000	0.664
MMseqs2	0.775	0.629
Synthetic (6XPF)	0.723	0.537
Synthetic (6XPD)	0.707	0.786

Table S9: TM-scores of the ZNT8 test case [4]. The AlphaFold2 experiments were computed on 6XPD|A residues 11-138 and 6XPF|A residues 4-131 due to unmodeled domains. We selected the domain with the biggest TM-score difference. Together with the backbone-lDDT score of Table S10 we argue that the prediction with MMseqs2 and Synthetic (6XPF) are on par with respect to 6XPF.

BB-lDDT	6XPF	6XPD
6XPF	1.000	0.719
MMseqs2	0.771	0.763
Synthetic (6XPF)	0.778	0.741
Synthetic (6XPD)	0.758	0.863

Table S10: Backbone lDDT of the ZNT8 test case [4]. The AlphaFold2 experiments were computed on 6XPD|A residues 11-138 and 6XPF|A residues 4-131 due to unmodeled domains. We selected the domain with the biggest TM-score difference. Backbone lDDT was chosen because for 6XPF only the backbone has been deposited.

<table>
<thead>
<tr><th>Type</th><th>ID (S)</th><th>ID (A)</th><th>Offset (S)</th><th>Offset (A)</th></tr>
</thead>
<tbody>
<tr><td>Fast-folding</td><td>1mhx|A</td><td>1ioj|A</td><td>8</td><td>0</td></tr>
<tr><td>Fast-folding</td><td>2f21|A</td><td>6e6n|A</td><td>0</td><td>38</td></tr>
<tr><td>Fast-folding</td><td>1vii|A</td><td>1k81|A</td><td>0</td><td>0</td></tr>
<tr><td>Fast-folding</td><td>2p6j|A</td><td>1ihv|A</td><td>0</td><td>0</td></tr>
<tr><td>Fast-folding</td><td>5awl|A</td><td>2nou|A</td><td>0</td><td>0</td></tr>
<tr><td>Fast-folding</td><td>2jof|A</td><td>7ald|A</td><td>0</td><td>0</td></tr>
<tr><td>Fast-folding</td><td>1fme|A</td><td>7mmy|A</td><td>0</td><td>0</td></tr>
<tr><td>Fast-folding</td><td>2hba|A</td><td>1fjk|A</td><td>0</td><td>0</td></tr>
<tr><td>Fast-folding</td><td>2wxc|A</td><td>2b9k|A</td><td>0</td><td>0</td></tr>
<tr><td>Fast-folding</td><td>1prb|A</td><td>1cad|A</td><td>0</td><td>0</td></tr>
<tr><td>Fast-folding</td><td>2a3d|A</td><td>5af5|A</td><td>0</td><td>0</td></tr>
<tr><td>Fast-folding</td><td>1lmb|4</td><td>3ncm|A</td><td>0</td><td>0</td></tr>
<tr><td>Diverse</td><td>1iad|A</td><td>1iqq|A</td><td>0</td><td>0</td></tr>
<tr><td>Diverse</td><td>1leo|A</td><td>3n0k|A</td><td>0</td><td>0</td></tr>
<tr><td>Diverse</td><td>1p6p|A</td><td>1acf|A</td><td>0</td><td>0</td></tr>
<tr><td>Diverse</td><td>1tzq|A</td><td>4ozs|A</td><td>0</td><td>0</td></tr>
<tr><td>Diverse</td><td>4etx|A</td><td>9epm|A</td><td>0</td><td>0</td></tr>
<tr><td>Diverse</td><td>4u3h|A</td><td>1eod|A</td><td>0</td><td>0</td></tr>
<tr><td>Diverse</td><td>8kc6|A</td><td>1p4x|A</td><td>0</td><td>0</td></tr>
<tr><td>Diverse</td><td>8bjc|B</td><td>1bf8|A</td><td>0</td><td>0</td></tr>
<tr><td>Diverse</td><td>7cri|A</td><td>2jf2|A</td><td>0</td><td>0</td></tr>
<tr><td>Diverse</td><td>4jm5|A</td><td>1prn|A</td><td>0</td><td>0</td></tr>
<tr><td>Diverse</td><td>6ufx|A</td><td>4pmy|A</td><td>0</td><td>0</td></tr>
<tr><td>Diverse</td><td>1cs6|A</td><td>6dfm|A</td><td>0</td><td>0</td></tr>
<tr><td>Diverse</td><td>4ee9|A</td><td>1f3l|A</td><td>0</td><td>0</td></tr>
<tr><td>Diverse</td><td>4e1s|A</td><td>1dxj|A</td><td>0</td><td>0</td></tr>
<tr><td>Diverse</td><td>1bsg|A</td><td>2of3|A</td><td>0</td><td>0</td></tr>
<tr><td>Diverse</td><td>5c1e|A</td><td>7rwf|A</td><td>0</td><td>0</td></tr>
<tr><td>Diverse</td><td>2hvm|A</td><td>8gok|A</td><td>0</td><td>0</td></tr>
<tr><td>Diverse</td><td>1muy|A</td><td>7tvn|A</td><td>0</td><td>0</td></tr>
<tr><td>Diverse</td><td>2e9u|A</td><td>1gci|A</td><td>0</td><td>0</td></tr>
<tr><td>Diverse</td><td>5ckm|A</td><td>1k0f|A</td><td>0</td><td>0</td></tr>
<tr><td>Diverse</td><td>2x8m|A</td><td>2p5y|A</td><td>0</td><td>0</td></tr>
<tr><td>Diverse</td><td>1doz|A</td><td>6ied|A</td><td>0</td><td>0</td></tr>
<tr><td>Diverse</td><td>2por|A</td><td>5fri|A</td><td>0</td><td>0</td></tr>
<tr><td>Diverse</td><td>1zxx|A</td><td>1qub|A</td><td>0</td><td>0</td></tr>
<tr><td>Diverse</td><td>3brx|A</td><td>1kkh|A</td><td>0</td><td>0</td></tr>
<tr><td>Diverse</td><td>1cps|A</td><td>4mag|A</td><td>0</td><td>0</td></tr>
<tr><td>Diverse</td><td>1bqc|A</td><td>7dg6|A</td><td>0</td><td>0</td></tr>
<tr><td>Diverse</td><td>6m0k|A</td><td>4hxx|A</td><td>0</td><td>0</td></tr>
<tr><td>Diverse</td><td>2hyv|A</td><td>7dme|A</td><td>0</td><td>0</td></tr>
<tr><td>Diverse</td><td>1que|A</td><td>7zya|A</td><td>0</td><td>0</td></tr>
</tbody>
</table>

Table S11: All selected (S) and adversary (A) proteins.

Selected (S)	Adversary (A)	TM S	TM A	pLDDT	Gap S	Gap A	Helix S	Sheet S	Helix A	Sheet A	Rel CO A	Rel CO S	CLASS S	CLASS A
1bsgA	2of3A	0.231	0.412	50.720	16.5	12.8	39.5	00.0	69.5	00.8	03.8	09.3	Alpha Beta	Mainly Alpha
2of3A	1bsgA	0.248	0.235	56.741	12.8	16.5	69.5	00.8	39.5	00.0	09.3	03.8	Mainly Alpha	Alpha Beta
1cpsA	4magA	0.260	0.771	59.830	21.2	11.1	35.8	00.7	43.6	00.0	09.1	08.6	Alpha Beta	Alpha Beta
4magA	1cpsA	0.256	0.545	49.417	11.1	21.2	43.6	00.0	35.8	00.7	08.6	09.1	Alpha Beta	Alpha Beta
1iqqA	1iadA	0.166	0.143	42.851	23.5	20.0	25.0	00.0	19.5	01.5	10.0	10.4	Alpha Beta	Alpha Beta
1iadA	1iqqA	0.306	0.664	52.527	20.0	23.5	19.5	01.5	25.0	00.0	10.4	10.0	Alpha Beta	Alpha Beta
3n0kA	1leoA	0.247	0.227	43.785	32.0	17.3	00.0	02.7	37.3	00.0	14.3	13.8	Mainly Beta	Alpha Beta
1leoA	3n0kA	0.157	0.161	42.360	17.3	32.0	37.3	00.0	00.0	02.7	13.8	14.3	Alpha Beta	Mainly Beta
3ncmA	1lmb4	0.417	0.329	59.200	17.4	16.3	04.3	00.0	64.1	00.0	07.5	19.7	Mainly Beta	Mainly Alpha
1lmb4	3ncmA	0.526	0.312	63.713	16.3	17.4	64.1	00.0	04.3	00.0	19.7	07.5	Mainly Alpha	Mainly Beta
1mhxA	1iojA	0.380	0.337	86.498	07.0	12.3	24.6	00.0	50.9	00.0	05.0	15.7	Alpha Beta	Few SS
1iojA	1mhxA	0.343	0.300	81.618	12.3	07.0	50.9	00.0	24.6	00.0	15.7	05.0	Few SS	Alpha Beta
1p6pA	1acfA	0.476	0.377	62.377	12.8	12.0	12.0	00.0	29.6	00.0	09.7	13.6	Mainly Beta	Alpha Beta
1acfA	1p6pA	0.253	0.254	47.387	12.0	12.8	29.6	00.0	12.0	00.0	13.6	09.7	Alpha Beta	Mainly Beta
1prbA	1cadA	0.718	0.284	96.831	13.2	28.3	60.4	00.0	00.0	07.5	18.1	14.2	Mainly Alpha	Mainly Beta
1cadA	1prbA	0.461	0.258	71.118	28.3	13.2	00.0	07.5	60.4	00.0	14.2	18.1	Mainly Beta	Mainly Alpha
1qubA	1zxxA	0.164	0.773	61.087	30.1	19.7	01.3	04.1	40.8	00.0	08.4	03.5	Mainly Beta	Alpha Beta
1zxxA	1qubA	0.221	0.193	45.222	19.7	30.1	40.8	00.0	01.3	04.1	03.5	08.4	Alpha Beta	Mainly Beta
1gciA	2e9uA	0.220	0.578	46.985	22.7	19.0	30.5	03.3	27.1	01.9	05.7	12.6	Alpha Beta	Mainly Alpha
2e9uA	1gciA	0.239	0.476	41.111	19.0	22.7	27.1	01.9	30.5	03.3	12.6	05.7	Mainly Alpha	Alpha Beta
2hbaA	1fjkA	0.249	0.510	78.097	17.3	05.8	26.9	03.8	80.8	00.0	04.5	13.5	Alpha Beta	Mainly Alpha
1fjkA	2hbaA	0.472	0.250	80.790	05.8	17.3	80.8	00.0	26.9	03.8	13.5	04.5	Mainly Alpha	Alpha Beta
2p6jA	1ihvA	0.704	0.294	87.972	21.2	13.5	59.6	00.0	00.0	00.0	19.9	12.5	Mainly Alpha	Mainly Beta
1ihvA	2p6jA	0.579	0.332	68.522	13.5	21.2	00.0	00.0	59.6	00.0	12.5	19.9	Mainly Beta	Mainly Alpha
2porA	5friA	0.250	0.528	44.996	14.0	21.6	05.6	02.0	30.9	02.7	06.0	08.4	Mainly Beta	Mainly Alpha
5friA	2porA	0.273	0.879	68.696	21.6	14.0	30.9	02.7	05.6	02.0	08.4	06.0	Mainly Alpha	Mainly Beta
2wxcA	2b9kA	0.670	0.218	77.636	29.8	23.4	42.6	00.0	00.0	00.0	25.1	12.6	Few SS	Mainly Beta
2b9kA	2wxcA	0.339	0.244	62.545	23.4	29.8	00.0	00.0	42.6	00.0	12.6	25.1	Mainly Beta	Few SS
2p5yA	2x8mA	0.169	0.177	39.382	23.5	26.4	39.5	01.0	00.0	05.1	03.6	07.9	Alpha Beta	Mainly Beta
2x8mA	2p5yA	0.208	0.724	57.472	26.4	23.5	00.0	05.1	39.5	01.0	07.9	03.6	Mainly Beta	Alpha Beta
1kkhA	3brxA	0.241	0.210	53.022	14.5	16.4	38.8	00.3	69.4	00.0	05.7	08.8	Alpha Beta	Mainly Alpha
3brxA	1kkhA	0.248	0.422	47.193	16.4	14.5	69.4	00.0	38.8	00.3	08.8	05.7	Mainly Alpha	Alpha Beta
4e1sA	1dxjA	0.242	0.686	49.493	12.8	25.6	04.5	00.8	41.7	02.1	08.7	12.2	Mainly Beta	Mainly Alpha
1dxjA	4e1sA	0.261	0.866	66.329	25.6	12.8	41.7	02.1	04.5	00.8	12.2	08.7	Mainly Alpha	Mainly Beta
1f3lA	4ee9A	0.230	0.356	51.219	15.0	22.1	20.2	02.5	33.3	02.2	07.7	08.1	Mainly Beta	Alpha Beta
4ee9A	1f3lA	0.256	0.668	48.933	22.1	15.0	33.3	02.2	20.2	02.5	08.1	07.7	Alpha Beta	Mainly Beta
4jm5A	1prnA	0.272	0.788	62.059	21.8	17.6	46.4	01.4	01.4	01.0	08.5	09.6	Mainly Alpha	Mainly Beta
1prnA	4jm5A	0.264	0.786	58.418	17.6	21.8	01.4	01.0	46.4	01.4	09.6	08.5	Mainly Beta	Mainly Alpha
1eodA	4u3hA	0.245	0.347	49.880	14.0	32.0	39.0	00.0	00.0	00.0	16.1	09.3	Alpha Beta	Mainly Beta
4u3hA	1eodA	0.757	0.254	71.180	32.0	14.0	00.0	00.0	39.0	00.0	09.3	16.1	Mainly Beta	Alpha Beta
5ckmA	1k0fA	0.173	0.796	63.517	28.9	14.4	00.0	01.8	41.9	00.7	07.2	08.4	Mainly Beta	Alpha Beta
1k0fA	5ckmA	0.295	0.343	44.477	14.4	28.9	41.9	00.7	00.0	01.8	08.4	07.2	Alpha Beta	Mainly Beta

Table S12: Scores of all *selected* (S) and *adversary* (A) proteins comparing the prediction of the *selected* input sequence and the inverse-folded MSA from the *adversary* compared to the two input structures. Next are relative gap, helix and sheet percentage of input structures as determined by DSSP [6]. Relative contact order (CO) [9] and CATH [8] classes of input structures.

**Selected (S)**	**Dataset (S)**	**Adversary (A)**	**TM-Score (S)**	**TM-Score (A)**	**pLDDT**
1zxxA	Mostly alpha	1p6pA	0.360	0.302	47.780
		3n0kA	0.306	0.252	47.790
		4u3hA	0.296	0.249	53.229
2e9uA	Mostly alpha	1p6pA	0.282	0.259	45.650
		3n0kA	0.173	0.168	42.654
		4u3hA	0.245	0.318	53.246
2p5yA	Mostly alpha	1p6pA	0.217	0.246	45.408
		3n0kA	0.197	0.281	43.153
		4u3hA	0.218	0.278	53.159
1prnA	Mostly beta	1acfA	0.334	0.555	47.150
		1leoA	0.204	0.260	45.905
		2of3A	0.241	0.565	59.006
2porA	Mostly beta	1acfA	0.268	0.253	48.112
		1leoA	0.208	0.271	45.683
		2of3A	0.235	0.156	48.998
4magA	Mostly beta	1acfA	0.176	0.309	46.196
		1leoA	0.233	0.181	48.121
		2of3A	0.233	0.278	56.759

Table S13: Table of attempts to make *selected* proteins (S), that failed before adapt another conformation with *adversarial* proteins (A) that worked previously. The *adversaries* were cut evenly at both ends to exactly match the size of the *selected* protein. Predictions of the input sequence of the *selected* protein with an inverse folded MSA from the *adversaries* were TM-scored [12] against the input *selected* protein (S) and input *adversary* protein (A) and the pLDDT of the prediction is reported.

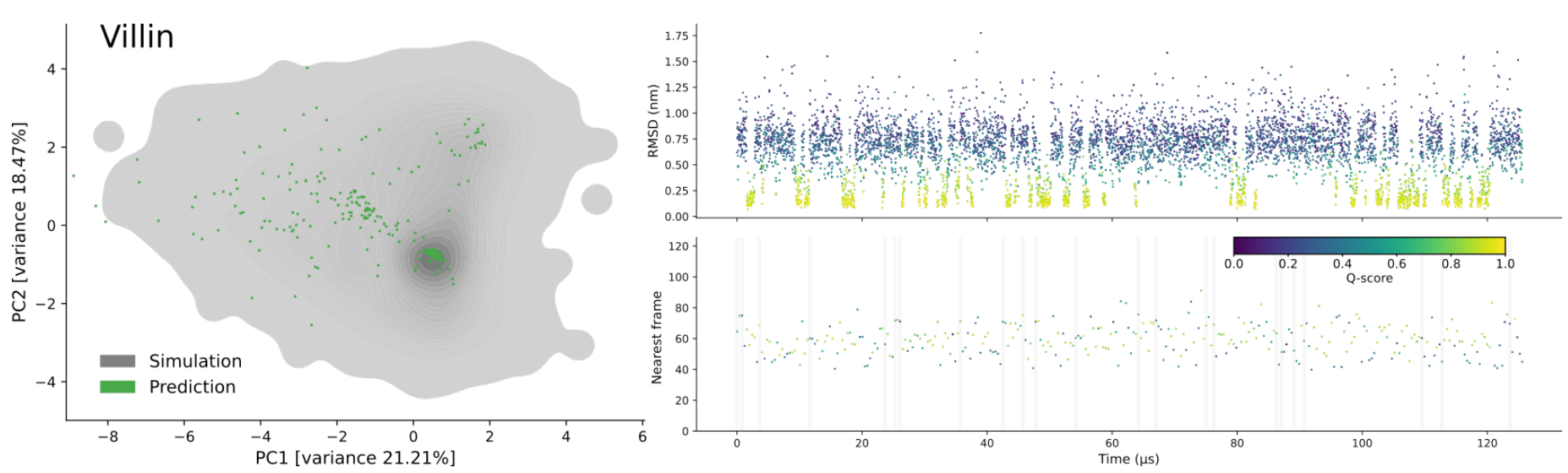

Figure S4: Conformational landscape from MD simulations of Villin compared to AlphaFold 2 predictions. Projection of simulation frames onto the eigenspace defined by the first two principal components (Left). Frame densities are represented as contour levels, with higher densities corresponding to more frequently sampled conformations. Green points indicate AlphaFold 2 predictions generated using frame-specific synthetic MSAs derived from the corresponding simulation frames. (Right) Timeline plots display simulation time on the x-axis, with RMSD to the experimental structure shown above and the index of the closest MD frame to each prediction shown below. To reduce noise, the average of the 20 nearest simulation frames is plotted. Predictions with fewer than five MD frames within 0.5 nm Cα RMSD are marked with vertical gray bars. Point colouring represents the Q-score relative to the experimental structure.

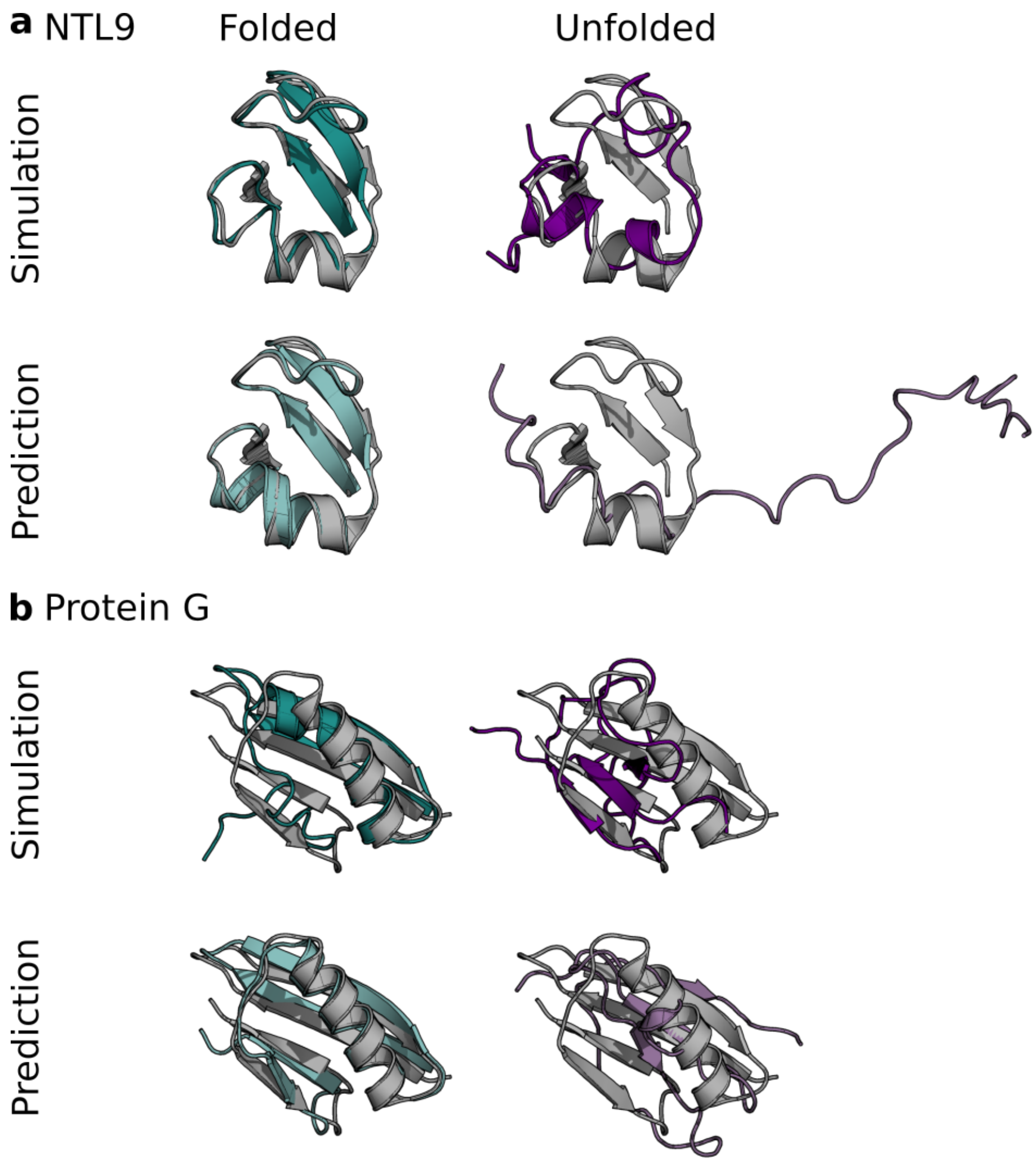

Figure S5: Example folded (left) and unfolded (right) structures from the simulation (top) and prediction (bottom) using our pipeline and the simulation frame as input of NTL9 (**a**) and Protein G (**b**). The experimental structure is in gray for comparison. Since it is hard to find a properly folded and unfolded structure for Villin, this protein is omitted.

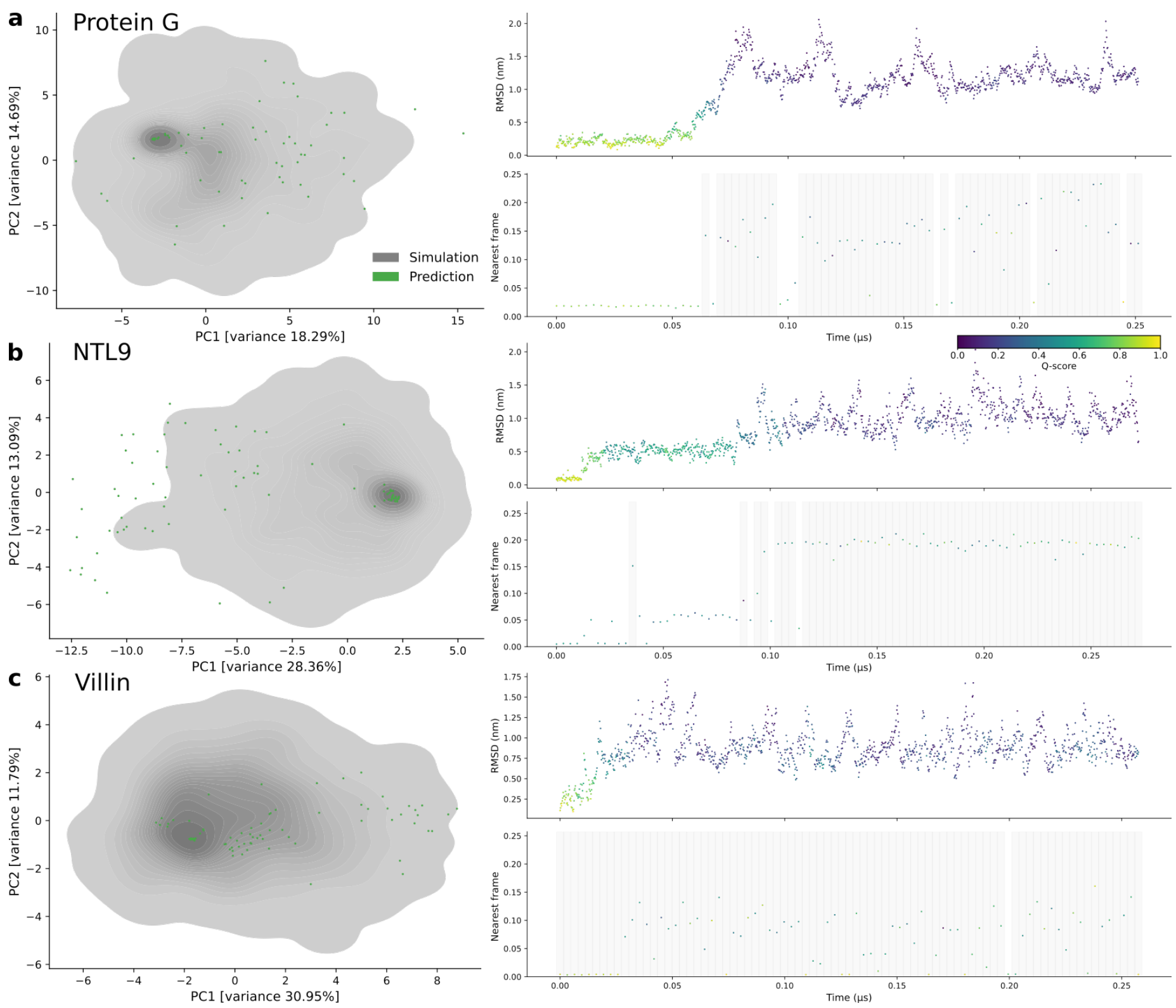

Figure S6: Conformational landscape from high temperature unfolding MD simulations of Protein G (**a**), NTL9 (**b**), and Villin (**c**) compared to AlphaFold 2 predictions. Projection of simulation frames onto the eigenspace defined by the first two principal components (Left). Frame densities are represented as contour levels, with higher densities corresponding to more frequently sampled conformations. Green points indicate AlphaFold 2 predictions generated using frame-specific synthetic MSAs derived from the corresponding simulation frames. (Right) Timeline plots display simulation time on the x-axis, with RMSD to the experimental structure shown above and the index of the closest MD frame to each prediction shown below. To reduce noise, the average of the 20 nearest simulation frames is plotted. Predictions with fewer than five MD frames within 0.5 nm Cα RMSD are marked with vertical gray bars. Point colouring represents the Q-score relative to the experimental structure.

FrankenMSA recipe

For the *Bombyx mori* Pheromone Binding Protein, the MMseqs2 MSA was generated by querying the ColabFold server for the full 2FJY|A input sequence and the synthetic MSA was generated using the full 2FJY|A structure. The MSA consisted from residue 0-16, 55-83 and 123-133 of the synthetic MSA and the rest of the MMseqs2 MSA.

Similarly, for the RSV Fusion glycoprotein F0, the MMseqs2 MSA was generated by triplicating the MSA generated from residue 25-510 from 5U68|A, which corresponds to the full input sequence without the unmodeled parts, followed by last 64 residues of UniProt P03420, which corresponds to the the transmembrane domain. The synthetic MSA was generated by inverse folding 5U68|A. To generate the FrankenMSA input, residues 0-100 are synthetic, while from 100-135, the residues are from MMseqs2, since there is an unmodeled part in the structure, then from 135-486 the sequences are synthetic again until the transmembrane domain from 486-550 is again generated using MMseqs2.

	**Apo**	**Holo**
**Apo**	1.000	0.694
**MMseqs2**	0.692	0.962
**FrankenMSA**	0.916	0.722

Table S14: TM-scores of *Bombyx mori* Pheromone-binding protein comparing MMseqs2 and FrankenMSA based AlphaFold 2 predictions with apo (2FJY) and holo (2P71) state.

	**Pre-fusion**	**Post-fusion**
**Pre-fusion**	1.000	0.483
**MMseqs2**	0.673	0.988
**FrankenMSA**	0.954	0.596

Table S15: TM-scores of three main chains of RSV Fusion glycoprotein F0 comparing MMseqs2 and FrankenMSA based AlphaFold 3 predictions with pre-fusion (5U68|ABC) and post-fusion (3RRR|BDF) state.

	**Pre-fusion**	**Post-fusion**
**Pre-fusion**	1.000	0.505
**MMseqs2**	0.685	0.956
**FrankenMSA**	0.958	0.636

Table S16: TM-scores of first chain of RSV Fusion glycoprotein F0 comparing MMseqs2 and FrankenMSA based AlphaFold 3 predictions with pre-fusion (5U68|A) and post-fusion (3RRR|B) state.

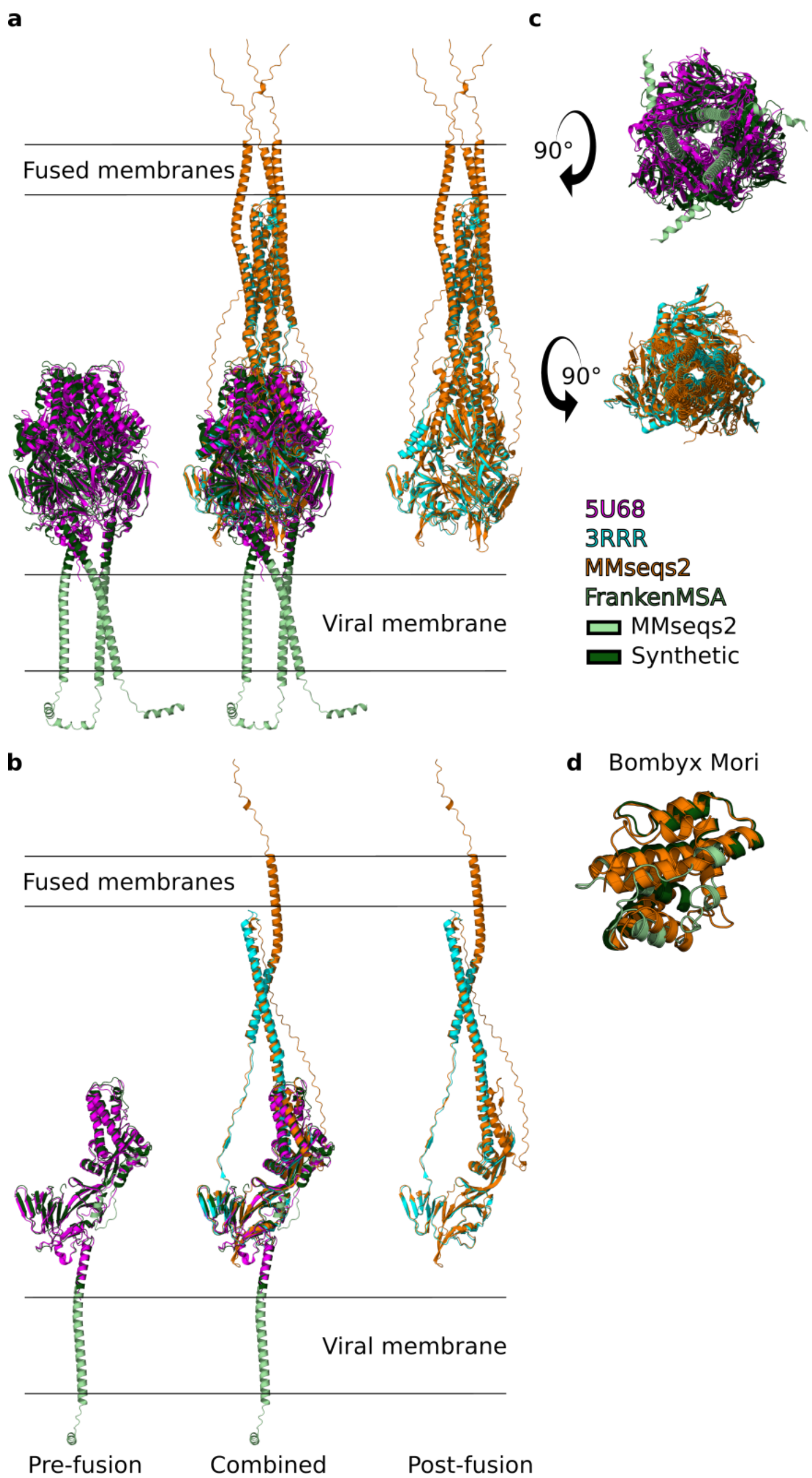

Figure S7: Additional FrankenMSA plots. RSV Fusion glycoprotein F0 plots comparing FrankenMSA AlphaFold 3 predictions (green, dark green for inverse folded, synthetic parts, light green for MMseqs2), MMseqs2 (orange), the experimental pre-fusion state (5U68, purple), and the experimental post-fusion state (3RRR, light blue). Showing the trimer (**a**), the first chain (**b**) and a view opposing their respective membrane (**c**). **d** shows a comparison of the *Bombyx mori* Pheromone Binding Protein FrankenMSA and MMseqs2 predictions.

References

[1] Bruce Alberts, Rebecca Heald, Alexander Johnson, David Morgan, Martin Raff, Keith Roberts, and Peter Walter. *Molecular biology of the cell: seventh international student edition with registration card*. WW Norton & Company, 2022.

[2] Devlina Chakravarty, Joseph W Schafer, Ethan A Chen, Joseph F Thole, Leslie A Ronish, Myeongsang Lee, and Lauren L Porter. Alphafold predictions of fold-switched conformations are driven by structure memorization. *Nature communications*, 15(1):7296, 2024.

[3] David M Corey, William P Dunlap, and Michael J Burke. Averaging correlations: Expected values and bias in combined pearson rs and fisher's z transformations. *The Journal of general psychology*, 125(3):245–261, 1998.

[4] Diego Del Alamo, Davide Sala, Hassane S Mchaourab, and Jens Meiler. Sampling alternative conformational states of transporters and receptors with alphafold2. *Elife*, 11:e75751, 2022.

[5] Steven Henikoff and Jorja G Henikoff. Amino acid substitution matrices from protein blocks. *Proceedings of the National Academy of Sciences*, 89(22):10915–10919, 1992.

[6] Robbie P Joosten, Tim AH Te Beek, Elmar Krieger, Maarten L Hekkelman, Rob WW Hooft, Reinhard Schneider, Chris Sander, and Gert Vriend. A series of pdb related databases for everyday needs. *Nucleic acids research*, 39(suppl_1):D411–D419, 2010.

[7] Faruck Morcos, Andrea Pagnani, Bryan Lunt, Arianna Bertolino, Debora S Marks, Chris Sander, Riccardo Zecchina, José N Onuchic, Terence Hwa, and Martin Weigt. Direct-coupling analysis of residue coevolution captures native contacts across many protein families. *Proceedings of the National Academy of Sciences*, 108(49):E1293–E1301, 2011.

[8] Christine A Orengo, Alex D Michie, Susan Jones, David T Jones, Mark B Swindells, and Janet M Thornton. Cath–a hierarchic classification of protein domain structures. *Structure*, 5(8):1093–1109, 1997.

[9] Kevin W Plaxco, Kim T Simons, and David Baker. Contact order, transition state placement and the refolding rates of single domain proteins. *Journal of molecular biology*, 277(4):985–994, 1998.

[10] Lauren L Porter and Loren L Looger. Extant fold-switching proteins are widespread. *Proceedings of the National Academy of Sciences*, 115(23):5968–5973, 2018.

[11] Charles Spearman. The proof and measurement of association between two things. 1961.

[12] Yang Zhang and Jeffrey Skolnick. Tm-align: a protein structure alignment algorithm based on the tm-score. *Nucleic acids research*, 33(7):2302–2309, 2005.
